# Spatial variability in sustainable development trajectories in South Africa: provincial level safe and just operating spaces

**DOI:** 10.1007/s11625-016-0418-9

**Published:** 2017-02-07

**Authors:** Megan J. Cole, Richard M. Bailey, Mark G. New

**Affiliations:** 1 School of Geography and the Environment, University of Oxford, South Parks Road, Oxford, OX1 3QY UK; 2 African Climate and Development Initiative, Geological Sciences Building, University of Cape Town, Rondebosch, 7701 South Africa; 3 School of International Development, University of East Anglia, Norwich Research Park, Norwich, NR4 7TJ UK

**Keywords:** Sustainable development, Sustainable development goals, Planetary boundaries, South Africa, Disaggregation

## Abstract

The Sustainable Development Goals (SDGs) represents the first globally agreed framework to address human development and environmental stewardship in an integrated way. One approach to summarising national SDG status is our “barometer for inclusive sustainable development in South Africa”. The barometer downscales global social and planetary boundaries to provide status and trends for 20 critical indicators of environmental stress and social deprivation. In this paper, we explore the sub-national heterogeneity in sustainable development indicators by creating barometers defining the ‘safe and just operating space’ for South Africa’s nine provinces. Our results show that environmental stress varies significantly and provinces need to focus on quite different issues. Although generally environmental stress is increasing, there are areas where it is decreasing, most notably, marine harvesting. Social deprivation results show more of a pattern with high levels of deprivation in employment, income and safety across the provinces, and historically disadvantaged provinces showing the most deprivation overall. Although deprivation is generally decreasing, there are notable exceptions such as food security in six provinces. Our provincial barometers and trend plots are novel in that they present comparable environmental and social data on key indicators over time for all South Africa’s provinces. They are visual tools that communicate the range of key challenges and risks that provincial governments face, and are non-specialist and accessible to a range of audiences. In addition, the paper provides a critical case study of spatial disaggregation of national data that is required for the SDGs implementation.

**Electronic supplementary material:**

The online version of this article (doi:10.1007/s11625-016-0418-9) contains supplementary material, which is available to authorized users.

## Introduction

Environmental sustainability, poverty eradication and reducing inequality pose continuing challenges for African countries in the twenty-first century. Population growth and global environmental change are expected to strain natural resources even further creating an urgent need to solve sustainability challenges across the continent (Gasparatos et al. 2016). The adoption in late 2015 of the ‘2030 Agenda for Sustainable Development’ and its 17 Sustainable Development Goals (SDGs) is the first time all nations have agreed to a ‘broad and universal policy agenda’ that addresses environmental, social and economic issues together (UN General Assembly 2015). The SDGs build upon the Millennium Development Goals (MDGs), but importantly for Africa, many African governments and civil society organisations were closely involved in the process of defining the SDGs. In addition, the 169 targets include means of implementation, i.e. finance, capacity building and technology transfer to developing countries. With over 230 global indicators (IAEG-SDG 2015) and many more national indicators to be developed, there is a need for tools to summarise and communicate progress on the SDGs and highlight national priorities. Some initial attempts have already been made with a SDG index for OECD countries (Kroll 2015), a regional SDG scorecard (Nicolai et al. 2015) and a SDG Index and Dashboard for all countries (Sachs et al. 2016).

In 2014 we developed and described a ‘national barometer for inclusive sustainable development for South Africa’ to propose a manageable set of national-level indicators and boundaries that are relevant in the South African context (Cole et al. 2014). Our barometer is based upon Rockström et al. (2009a, b) ‘planetary boundaries’ and Raworth’s (2012) ‘safe and just space’ framework, colloquially known as the Oxfam doughnut. The planetary boundaries are a set of nine critical global environmental indicators plotted against their safe environmental boundaries (based on critical thresholds and unacceptable levels of environmental stress) to highlight where excessive stress is occurring at the global scale. The ‘safe and just space’ framework adds 11 social indicators plotted against their global social foundation/floor (zero extreme deprivation) to the planetary boundaries. Together they define a social floor and an ‘environmental ceiling’ and provide a visual aspirational goal for achieving inclusive sustainable development. Using the same approach as Rockström et al. (2009a, b) and Raworth (2012), our barometer is visually presented in two radar plots. It shows how close South Africa is to its safe environmental boundaries (for climate change, ozone depletion, freshwater use, arable land use, phosphorus loading, nitrogen cycle, biodiversity loss, marine harvesting and air pollution) and what proportion of the population lives below the national social floor (for electricity access, water access, sanitation, housing, education, health care, voice, jobs, income, household goods, food security and safety).

There are strong arguments for using planetary boundary thinking at sub-global levels where policy action and natural resource management most commonly occur (Dearing et al. 2014; Fang et al. 2015; Steffen et al. 2015). Our barometer brings critical thresholds and scientifically informed environmental limits into the national policy-making discourse in a simple and relevant way. Unlike the studies for European countries that use the planetary boundary concept to determine their negative global environmental impact (Nykvist et al. 2013; European Comission 2014; Dao et al. 2015; Hajer et al. 2015) our study uses it as a warning light that exposes the risks that could hinder South Africa’s ability to meet its national development goals. This development focused approach, rather than the environmental limits thinking used in Europe, is relevant across Africa. As SI Table S1 shows, all of the indicators in our barometer have direct relevance to the SDGs, and could be used as SDG indicators.

While national level reporting is both informative and necessary, knowledge of sub-national heterogeneity in environmental stresses and social deprivation is important for strategic planning to deliver sustainable development. Disaggregation to finer scales would quantify sub-national states and boundaries, identify where the most pressing challenges are and where action is needed, and would better fit policy implementation scales. It would also contribute to SDG monitoring which requires data disaggregation to expose inequalities, encourage sub-national implementation and avoid perverse incentives and unequal progress (SDSN 2015a).

In this paper, we disaggregate our South African national barometer to the provincial level to explore the heterogeneity in national indicators and to provide a case study of disaggregation for SDG implementation. In our “Methodology” section, we provide details on three disaggregation approaches that we used for the environmental dimensions. In our “Results” section, we present the data in radar plots similar to the original planetary boundaries and social foundation. We also provide trend plots for the change in status over the past 20 years. In our “Discussion” section, we look at (a) sub-national variability in sustainable development indicators, (b) barometers as policy tools, (c) environmental governance and safe boundaries, (d) defining social floors, and (e) implementing the SDGs.

## Methodology

### South African context

South Africa has a diverse environment ranging from semi-desert to sub-tropical forest and exceptional biodiversity (Driver et al. 2012) making it one of 17 mega-diverse countries in the world (UNEP 2014). It is the 30th driest country in the world (DWA 2013) and only 12% of land is capable of supporting rain-fed crop production (Collett 2013). Climate change projections for South Africa show significant warming, as high as 5–8 °C over the interior by 2100, and a risk of drier conditions in the west and south, and wetter conditions in the east (DEA 2013a). The country has rich mineral deposits, including gold, platinum, iron ore, diamonds and coal. The mining sector has played a key role in the economy for 140 years, making South Africa the most industrialised country in Africa (Chamber of Mines of South Africa 2013). South Africa is also the biggest greenhouse gases (GHG) emitter, and is responsible for 38% of Africa’s carbon emissions (Boden et al. 2011).

Despite being the largest economy in Africa, roughly half of the population of 55 million live below the national upper-bound poverty line (DPME 2015a), and more than 10% of people live on less than $1.25 per day (DPME 2013). Over 38% of the labour force (including discouraged jobseekers) is unemployed (StatsSA 2016a) and South Africa’s labour force participation rate (58%) is among the lowest in Africa (World Bank 2016). South Africa has one of the world’s highest levels of income inequality (Palma 2011) with a Gini coefficient of 0.65 in 2010 (DPME 2015a). It has spatial inequality across multiple aspects of social deprivation (Wright and Noble 2009), a legacy of the racial segregation of Apartheid.

**Fig. 1:**
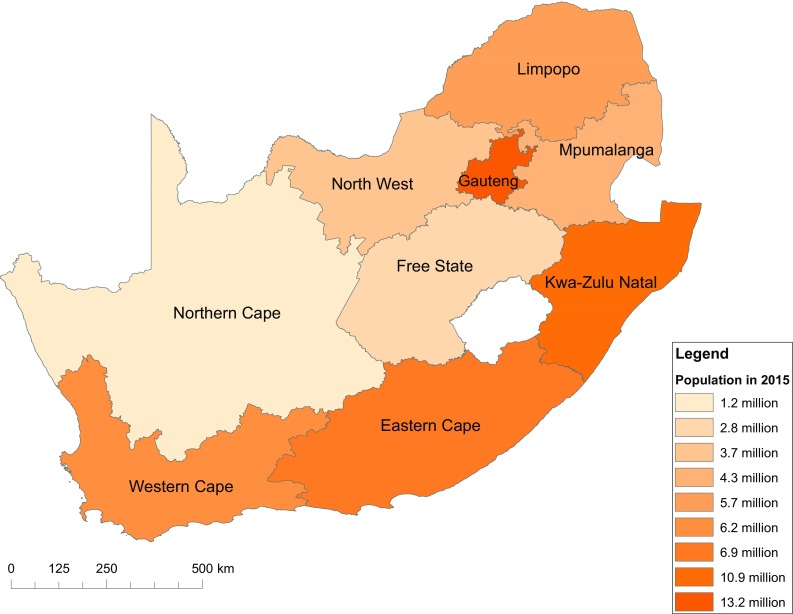
Map of the provinces of South Africa, *shaded* by population in 2015

South Africa has a unitary but decentralised state with cooperative governance between three spheres of government—national, provincial and local (Republic of South Africa 2012). The nine provinces—Eastern Cape, Free State, Gauteng, KwaZulu-Natal, Limpopo, Mpumalanga, Northern Cape, North West and Western Cape—were created as part of the transformation to democratic rule in 1994. They were based on a set of ‘development regions’ aimed at planning across previous racially based administrative boundaries and were given considerable functions in the Constitution (Wittenberg 2006). In 2015/16 the provinces received 43% of the national budget with significant autonomy to allocate resources to respond to provincial priorities and meet national objectives (National Treasury 2015). The provinces, therefore, have the mandate and in theory the ability to address many of the environmental and social challenges highlighted in our national barometer. The provinces are shown in Fig. 1 and summarised in Table 1.

**Table 1:** Population, area, population density and GDP of the provinces

Province	Population in 2015^a^		Area in square kilometres^b^	Population density in people per square kilometre	Percentage GDP in 2014^c^	Metropolitan areas
	Number	Percentage				
Eastern Cape	6,916,200	12.6	168,966	40.9	7.6	Nelson Mandela Bay, Buffalo City
Free State	2,817,900	5.1	129,825	21.7	5.0	Mangaung
Gauteng	13,200,300	24.0	18,178	726.2	34.4	Ekurhuleni, Johannesburg, Tshwane
KwaZulu-Natal	10,919,100	19.9	94,361	115.7	16.1	eThekwini
Limpopo	5,726,800	10.4	125,754	45.5	7.2	
Mpumalanga	4,283,900	7.8	76,495	56.0	7.5	
Northern Cape	1,185,600	2.2	372,889	3.2	2.1	
North West	3,707,000	6.7	104,882	35.3	6.6	
Western Cape	6,200,100	11.3	129,462	47.9	13.6	Cape Town
South Africa	54,956,900	100	1,220,813	45.0	100	

^a^Data source: Mid-year population estimates (StatsSA 2015b)

^b^Data source: Census 2011 (StatsSA 2012c)

^c^Data source: GDP Quarter 4 2015 (StatsSA 2016d)

### National barometer

In creating our national barometer, we developed a decision flowchart to assess the environmental and social dimensions, indicators and boundaries that make up the ‘safe and just operating space’ and adapt them to the national level. The aim was to ensure repeatability and consistency so that it could be used in other countries or at other scales (Cole et al. 2014).

The criteria used for selecting dimensions were ‘Is this relevant at the national scale?’ and ‘Does the set of dimensions include the main environmental and social concerns in South Africa?’. The criteria for indicator selection were (a) ‘Is the indicator the best available direct measure of that dimension?’, ‘(b) Are there sufficient reliable data that are measured on a regular basis?’ and (c) ‘Can a national boundary be determined?’ If the existing global dimension or indicator did not meet the criteria then it was removed or replaced with a more appropriate national-scale choice. These criteria are similar to the proposed criteria for SDG indicators, which should be relevant, methodologically sound, measurable, easy to communicate and access, limited in number and outcome-focused (UNSD 2015a). The data were taken from relevant national databases and reports, international databases and academic papers. We also sought expert judgment on indicators and boundaries through semi-structured interviews with 43 South African experts from national, provincial and metropolitan government, national research institutes, universities and international NGOs.

To create the provincial barometers, we did not use the decision flowchart to select new indicators, as we wanted to explore sub-national heterogeneity in those indicators we had already chosen. Instead we used three methods of disaggregation of national data for the dimensions in our national barometer: (a) share the national total amongst the provinces, (b) aggregate local data to the provincial scale, and (c) fit data reported by ecological units into administrative borders. These methods are described further below.

We updated the data sources where new data were available, or where sub-national data sources could be found. The data were used to produce nine provincial barometers for both environmental stress and social deprivation. We also plotted the average annual change since 1994 (or since data collection for each specific indicator began) for all the dimensions in two graphs. We did not plot the yearly status due to space constraints, as it would require 20 graphs.

### Environmental stress

In our national barometer, we used the Environmental Sustainability Indicators (ESI) technical report (DEA 2013b) published annually by the Department of Environmental Affairs (DEA) as a starting point for our analysis. The ESI was developed based on a comprehensive review of potential national indicators, Yale’s Environmental Performance Index (Hsu et al. 2016) and the DPSIR framework (e.g. Hammond et al. 1995; Gabrielsen and Bosch 2003). We then reviewed relevant national policies, reports and assessments, and academic literature to identify the most suitable dimensions, indicators and boundaries and tested these with experts. While we adapted three of Rockström et al’s (2009a, b) dimensions we adjusted all of the indicators and boundaries to suit national scale and circumstances. For the provincial barometers, we reviewed the most recent provincial State of Environment and State of Biodiversity reports.

Table 2 shows the environmental dimensions, indicators, data sources, level of confidence, and the method of disaggregation used in the provincial barometers. Table 2 also shows the type of safe environmental boundary for each dimension, as defined in our national barometer. Type A is an internationally agreed target based on a global biophysical threshold, which varies by country based on differences in national capability and responsibility. Type B is a national biophysical limit for the sustainable use of land or freshwater resources, which can include or exclude human intervention such as infrastructure and technology, and uses local biophysical thresholds to define the boundary. Type C is a local biophysical threshold based on established research and expert judgment in the country being studied, and is unaffected by scale (i.e. national and provincial boundaries are the same). Each dimension is briefly explained below with further details given in the SI.

**Table 2:** Dimensions for environmental stress for the provincial barometers

Dimension	Indicator (units in brackets)	State				Boundary		
		Data source	Year	Disaggregation method	Level of confidence	Data source	Type	Disaggregation method
Climate change	Annual direct CO_2_ emissions (MtCO_2_)	United Nations (DEA), StatsSA	2011	Share national total	Medium	Long term mitigation scenarios	Type A	Share national total
Ozone depletion	Annual HCFC consumption (ODPt)	United Nations (DEA), HCFC phase-out plan	2015	Aggregate local data	High	HCFC phase-out plan		Aggregate local data
Freshwater use	Annual consumption of available freshwater resources (Mm^3^/a)	Reconciliation strategies (DWS)	2011	Aggregate local data	Medium	Reconciliation strategies	Type B	Aggregate local data
Arable land use	Arable land converted to cropland (ha)	Preservation and development of agricultural land framework bill (DAFF)	2013	Provincial data exists	High	Preservation and development of agricultural land framework bill		Provincial data exists
Nutrient cycle	Total Phosphorus concentration in freshwater (mg/l)	National Eutrophication Monitoring Programme (DWS)	2012	Aggregate local data, fit borders	Medium	Oberholster and Ashton 2008	Type C	Not affected by scale—same as national
	Nitrogen application rate (kgN/ha)	Not available				Brentrup and Palliere (2010)		
Biodiversity loss	Endangered and critically endangered ecosystems (%)	National biodiversity assessment (DEA)	2011	Fit borders, aggregate local data	Medium	Expert judgment		
Marine harvesting	Depleted marine fisheries stocks (%)	Status of marine fishery resources (DAFF)	2013	Fit borders	Low	Expert judgment		
Air pollution	Annual average PM10 concentration (µg/m^3^)	State of air report (DEA)	2014	Aggregate local data	High	State of air report		
Chemical pollution	To be determined							

*DEA* Department of Environmental Affairs, *DWS* Department of Water and Sanitation, *DAFF* Department of Agriculture, Forestry and Fisheries, *StatsSA* Statistics South Africa

#### Climate Change

Rockström et al. (2009a) based their climate change indicator and boundary on global atmospheric carbon dioxide (CO_2_) concentrations. As this cannot be disaggregated to the national level, we used CO_2_ emissions for our national indicator. Our safe boundary is based on the emissions trajectory of the ‘Required by Science’ scenario in the Long Term Mitigation Scenarios, LTMS (Scenario Building Team 2007), which South Africa uses for its national commitments to the United Nations Framework Convention on Climate Change. In 2011 South Africa emitted 477.7 MtCO_2_ (UNSD 2015b) and the safe boundary is calculated as 453.7 MtCO_2_. South Africa’s national inventory (DEAT 2009) reports sub-national data by sector, not by region, with only four provinces having their own emissions inventories (but only for different years) (Gauteng 2007, Eastern Cape 2008, Western Cape 2009, Free State 2012).

For the provincial status we, therefore, had to share national CO_2_ emissions for the status and boundary between provinces. As a province’s share of the population can be quite different to its energy use, it would not be equitable to use population as the basis for disaggregation. Instead we used provincial electricity consumption (StatsSA 2012a) to allocate provincial emissions (see Table S3 in the SI) as it has the largest share (46%) of national CO_2_ emissions. We used consumption (and not production) as it is reported at provincial level. Although this overestimates CO_2_ emissions in provinces with low carbon energy sources such as wind and nuclear, we did not have the necessary data to adjust the figures. Our calculated figures correlate reasonably well with the four provincial inventories that are available (see SI). We shared the national boundary using the provincial contribution to GDP (StatsSA 2012b) (see SI Table S3) to measure the energy intensity and thus mitigation responsibility of each provincial economy. To analyse the trends we used the year 2002 as this was the furthest back we could obtain electricity use by province (StatsSA 2002). As the LTMS baseline year is 2003 there is no ‘required by science’ target for the year 2002, hence we shared the actual national emissions of 347.7 MtCO_2_ between the provinces (see SI Table S4).

#### Ozone depletion

Rockström et al. (2009b) based their ozone depletion indicator and boundary on the global ozone concentration. As this cannot be disaggregated to the national level, we used consumption of hydro-chloro-fluoro-carbons (HCFCs) for our national barometer. In line with the Montreal Protocol, South Africa has phased out the production and consumption of all ozone-depleting (ODP) substances except HCFCs (DEA 2014a) and is a consumer rather than a producer of HCFCs. For the provincial status, we aggregated individual company HCFC-22 and HCFC-141b consumption data for 2010 (NEDLAC 2012). We then projected it to 2015 based on the latest national HCFC consumption figure of 238.6 ODPt reported by the UNEP Ozone Secretariat (UNEP 2016) (see SI Table S5).

This showed that distributors in Gauteng, Western Cape and KwaZulu-Natal consume all the HCFCs. The national boundary is based on the government commitment to reduce HCFC consumption to 332.7 ODP tonnes by 2015 and eliminate it by 2040 (NEDLAC 2012). We shared this between these three provinces based on their share of HCFC consumption. As historical sub-national data do not exist, for the trend analysis we used the 2010 provincial ratios of HCFC consumption to share the 103.3 ODPt of HCFCs consumed in 1990 (UNEP 2016). We used the government target to freeze consumption at 370 ODPt in 2013 as no limits are defined before 2013.

#### Freshwater use

Rockström et al. (2009a) measured the consumption of freshwater by humans, the global aggregate of local use. In our national barometer we used South Africa’s freshwater consumption reported in the National Water Resource Strategies (DWAF 2004; DWA 2013). Our safe boundary was the available water supply, which takes ecological requirements into account. For the provincial barometers, we could use the demand and supply of the 19 Water Management Areas and 87 sub-areas (see SI Table S6), however, these figures are only available for the year 2000. We considered using the government’s current Water Allocation Registration Management System (WARMS) database, but this would only provide water allocation not demand and supply.

We decided to use demand and supply figures found in the Department of Water and Sanitation’s (DWS) 840 reconciliation strategies for all towns in 2008 and Water Supply Systems that supply the metropolitan areas. The All Town Studies provide the first comprehensive water use information at the local level across South Africa and are aimed at informing water resource investment and management decisions (DWA 2013). As the reconciliation strategies do not account for ecological requirements, we reduced the supply using the ecological requirements for the year 2000 to provide a more accurate picture of the stress on freshwater supply (see SI Table S6). While this may overestimate the reserve as total supply includes groundwater, the reserve figures in 2000 did not include estuaries, which usually have higher ecological requirements (DWAF 2004). As the reconciliation strategies focus on domestic water demand, agriculture and heavy industry are not included in our results. This is not ideal but it is the best available dataset. In addition, annual progress reports are published for the Water Supply Systems and the town strategies are being updated, so more recent data will become available which will allow the calculation of long-term trends.

#### Arable land use

Rockström et al. (2009a) focused on land use change and its detrimental effects on biodiversity and climate change. However, South Africa’s land cover has remained relatively stable since 1961 (Niedertscheider et al. 2012; Schoeman et al. 2013). The only national land degradation study was done by Hoffman et al. in 1999 and is qualitative not quantitative (DEAT 2006). South Africa is largely a semi-arid country with very limited land capable of supporting sustainable crop production (Collett 2013). We therefore focused on land capability, i.e. the ‘total suitability for use, in an ecologically sustainable way, for crops, for grazing, for woodland and for wildlife… exclusive of social and economic variables’ (Schoeman et al. 2002). The national land capability classification defines eight classes based on a combination of climate, soil and terrain. Arable land (i.e. land that can be used for crop production) is termed ‘arable land of acceptable quality for crop production’ (Classes I-III) or ‘marginal arable land’ (Class IV).

Our indicator for land use is total arable land (Classes I–IV) converted to cropland and our safe boundary is acceptable arable land (Classes I–III). We excluded marginal arable land from the boundary as it is more prone to crop failures in low rainfall years (Biggs and Scholes 2002) and requires irrigation to be sustainable in the long-term. Data at the provincial level is available in the draft Preservation and Development of Agricultural Land Framework Bill (DAFF 2015) which improves on previous datasets as it measures cultivated land for each land capability class. We aggregated cultivated land for the status (Class I–IV) and boundary (Class I-III) (see SI Table S7). Cropland in the non-arable classes (Classes V–VIII) is termed ‘unique farmland’, e.g. Cape Winelands in Class IV and VI which can be sustainably farmed despite shallow natural soil depth (Collett 2013). As the specific figures for unique farmland are not provided we excluded it from the analysis, although this does mean that the Western Cape exceeds its boundary. We could not calculate the trend over time as the cultivated land per land capability class has not been reported before.

#### Phosphorus loading

Rockström et al. (2009a) argued that the additional phosphoros (P) and nitrogen (N) activated by humans is disturbing the global cycles. Eutrophication of freshwater resources is a global concern (Steffen et al. 2015) and is widespread in South Africa (van Ginkel 2011). South Africa’s National Eutrophication Monitoring Programme measures levels of chlorophyll and phosphorus at over 1,200 monitoring points in 16 drainage basins. In our national barometer we used mean annual total phosphorus (P) concentrations in freshwater as the indicator. We used South Africa’s critical threshold, and effluent discharge limit for wastewater treatment plants of 0.10 mg/l (Oberholster and Ashton 2008) for the safe boundary.

For the provincial barometers, we aggregated total P concentrations reported by drainage basin and calculated weighted averages using gross drainage basin volumes (DWA 2014). We then matched basins to provinces so that each province was an average of weighted total *P* values (see SI Table S8). Where basins were shared by provinces, we included them in all the relevant provinces. We used the national boundary for all provinces as it is a local threshold. We calculated the trend from 2000 to 2012 using the same dataset and boundary.

#### Nitrogen cycle

Nitrogen is essential for food production. However, nitrogen fertiliser use can have a range of local negative effects (Rockström et al. 2009b; de Vries et al. 2013). Sustainable fertiliser use for crop production can be measured using the nitrogen balance or the nitrogen use efficiency (Brentrup and Palliere 2010). Both indicators are calculated using nitrogen (N) applied to the soil through fertilisers and nitrogen removed from the soil by crop production. In our national barometer we used the nitrogen use efficiency (N removed divided by N applied) in maize production, which uses 62% of all nitrogen in fertiliser in the country (FertASA 2013). Sub-national data on fertiliser consumption for maize or any other crop is not available. Sharing the national total between the provinces by crop area or yield would not take variations in soil and climate into account. We, therefore, could not populate this indicator for the provinces.

#### Biodiversity loss

Rockström et al. (2009a) measured the extinction rate of species, which saw a massive acceleration in the twentieth century. In 2004 the South African National Biodiversity Institute (SANBI) started to assess biodiversity by ecosystem, rather than species, threat status. The methodology was improved in 2011 and we used the percentage of critically endangered (CR) and endangered (EN) ecosystems for our national biodiversity loss indicator. Our safe boundary was that no ecosystems should be endangered or critically endangered.

For the provincial status, each ecosystem type required a slightly different approach. Estuarine ecosystems were reported at district level (van Niekerk and Turple 2012) and had to be aggregated. Inshore marine and coastal ecosystems were reported by habitat type and geographic region (Sink et al. 2012) and had to be matched to the four coastal provinces. Terrestrial ecosystems were reported by province (DEA 2011). Interviews with experts at SANBI suggested we convert each total area to a percentage and average the three ecosystem types by area to obtain a single value for percentage CR and EN ecosystems per province (see SI Table S10). We kept the safe provincial boundary the same as the national boundary. We did not determine the threat status for freshwater ecosystems (rivers and wetlands) as they are reported by the old 19 Water Management Areas (Nel and Driver 2012), which do not match well to the provinces. We could not calculate trends as the methodology changed from 2004 to 2011.

#### Marine harvesting

In our national barometer we replaced Rockström et al.’s (2009a) ocean acidification with marine harvesting due to the lack of understanding of the process in South Africa’s marine environment (CSIR 2012). Our national indicator was depleted marine fisheries (below the biomass level at which maximum sustainable yield is obtained) and our safe boundary was zero depleted marine fisheries. Recently a new Ocean Acidification Indicator (ACID-I), defined as the aragonite saturation state, has been defined for the west coast of South Africa (DEA 2015a) but is not comprehensive enough to be used here. As marine harvesting is only relevant for the four coastal provinces (Eastern Cape, Western Cape, Northern Cape, KwaZulu-Natal) we considered changing the dimension to ‘aquatic harvesting’ to include inland fisheries. However, there are almost no data on inland harvesting rates or stock status (McCafferty et al. 2012). For marine harvesting at provincial level, we estimated the depleted status (percentage of total number of species with known status) per province based on the geographic location of the fisheries (DAFF 2014) (see SI Table S12). Our safe boundary is zero. We calculated the trend from 2009, when reporting started, to 2013, which is the most recent data.

#### Air pollution

In our national barometer, we replaced Rockström et al’s (2009a) atmospheric aerosol loading with the more relevant dimension air pollution. Particulate matter less than 10 microns (PM_10_) is the ‘greatest national cause for concern in terms of air quality’ and is used for the National Air Quality Indicator, NAQI (DEA 2013c, 2015b). Annual PM_10_ concentrations for monitoring stations in mining or industry hubs, coal-fired power stations and very large urban centres are reported in ‘State of the Air’ reports. We used this data and indicator in our national barometer. We used the national PM_10_ limit of 50 μg/m^3^ (DEA 2009) as our safe boundary.

For the provincial barometers, we aggregated the monitoring station data in the six provinces (Gauteng, KwaZulu-Natal, Limpopo, Mpumalanga, North West and Western Cape) used in the NAQI to determine provincial averages (see SI Table S13). The national PM_10_ limit decreased to 40 μg/m^3^ in 2015 (DEA 2014b) and we used this for our provincial boundaries. Although monitoring began in 1994, it was not comprehensive and we calculated the trend from 2003 to 2014 to ensure all relevant provinces were covered.

#### Chemical pollution

Similarly to Rockström et al. (2009a) and Steffen et al. (2015), we did not identify a national indicator for chemical pollution due to the lack of detailed and accurate data. Although South Africa’s National Waste Information Baseline Report (DEA 2012a) provides an estimated baseline, reporting is voluntary and measurement is incomplete.

### Social dimensions

To determine the 12 dimensions and indicators in our national barometer, we used the South African Index of Multiple Deprivation (SAIMD) (Noble et al. 2009; Wright and Noble 2009) and the annual Development Indicators report (DPME 2013), published by the South African Presidency. Both have been informed by international good practice and adapted to South African conditions and are used by the government on a regular basis. We made a number of changes to the original 11 Raworth (2012) dimensions. We separated water and sanitation into individual dimensions, we added housing, household goods and safety, and we removed resilience, social equity and gender equality. Expert interviews suggested that resilience is a cumulative effect that is dependent on the other dimensions, and therefore, an indirect measure. Experts also felt that both social equity and gender inequality should be incorporated into the other dimensions, as they are cross-cutting. Although social equality and gender equality have dedicated SDG goals (Goal 5 and Goal 10) they are mainstreamed throughout and will be covered by data disaggregation.

The social indicators in our barometer reflect national priorities and official indicators. The social floor (boundary) for each dimension is determined by the indicator selected and the goal that nobody (0% of the population) lives in deprivation. There is usually a set of indicators to choose from that reflects a range in social deprivation. The choice of indicator, therefore, partly determines the definition of the social floor.

There are three types of indicator sets that we identified. Type 1 indicators are typically reported as a range of levels of access, as are commonly found in household surveys. For example, choosing ‘access to piped water within 200 m of the dwelling’ rather than ‘access to piped water in the dwelling’ sets a lower social floor. Type 2 indicators have a range of definitions of the same broad indicator. For example, unemployment can be defined as narrow or broad, where the latter includes discouraged jobseekers. Type 3 indicators offer diverse representations of different aspects of a dimension. For example, material deprivation can be measured by ownership of a refrigerator, washing machine, radio and/or television.

We did not define an indicator for voice in the national barometer. This is because there is a lack of a generally accepted definition of voice, a lack of consensus among experts on a single indicator, as well as a large range in values for different indicators. Without other countries to compare it to, it would not have added much value. However, for the provincial barometers, we felt that it would be worthwhile to select an indicator for voice as the comparison between provinces can circumnavigate the problem of the variation in values for different indicators. Development Indicators 2012 lists four indicators under the heading ‘Social cohesion: Voice and Accountability’ that could measure voice: membership of voluntary organisations, voter turnout, female representation in parliament and the corruption perceptions index. None of these were used, however, based on expert judgment or because the indicator is not a deprivation measure or is gender-specific. The most appropriate indicators were found in the Afrobarometer, a comparative series of independent public attitude surveys on democracy and governance run since 1990 in 35 African countries (Citizen Surveys 2013). We identified 14 possible indicators, shown in SI Table S14. There is quite good correlation between the different indicators in terms of comparing the provinces. We chose the indicator ‘people who feel they are not free to say what they think’ as it is easy to understand and shows meaningful variation between provinces.

As all social data could be found at the provincial level in existing reports or databases, no special disaggregation methods was performed. Table 3 shows the 12 social dimensions, indicators and data sources in our provincial barometers, grouped into four domains—basic services, public goods, livelihoods and living standards. We largely used the 2015 General Household Survey, GHS (StatsSA 2016b), a key data source for Development Indicators, as it had the most recent data. For the indicators not covered by the GHS, we used the Development Indicators 2014 report database (DPME 2015b), the 2014/15 Victims of Crime Survey (StatsSA 2015a), the Quarterly Labour Force Survey Fourth Quarter 2015 (StatsSA 2016c), and the South African Afrobarometer Round 5 (Citizen Surveys 2013).

**Table 3:** Dimensions of social deprivation for the provincial barometers

Domain	Dimension	Indicator of deprivation (units all %)	Year	Data source	Indicator type
Basic services	Energy access	Households not connected to mains electricity	2015	General household survey 2015	Type 1
	Water access	Households without access to piped water within 200 m (≥RDP standard)	2013	Development indicators 2014	
	Sanitation	Households without a toilet or ventilated pit latrine	2015	General household survey 2015	
	Housing	Households without a formal dwelling	2015	General household survey 2015	
Public goods	Education	Adult illiteracy rate (population aged 15 years or older with education level lower than Grade 7)	2015	General household survey 2015	Type 3
	Health care	Infant (<1 year) immunisation coverage	2014	Development indicators 2014	
	Voice	People who feel they are not free to say what they think	2011	Afrobarometer 2011	
Livelihoods	Jobs	Broad unofficial unemployment rate (adults aged 15–64 available to work)	2015	Quarterly labour force survey quarter 4 2015	Type 2
	Income	Population living below the national poverty line (R577/month in 2011 Rands)	2011	Development indicators 2014	
Living standards	Household goods	Households that do not own a refrigerator	2015	General household survey 2015	Type 3
	Food security	Households without adequate food	2015	General household survey 2015	
	Safety	Households that feel unsafe walking alone in their area at night	2015	Victims of crime survey 2014/15	

To plot the trends in the social dimensions, we used the same data sources so that the figures are comparable, as sometimes other data sources used different calculation methodologies. We looked for data from 1994 or similar, as we had done in the national barometer. However, we found that the Development Indicators 2014 generally reported provincial data from 2001 onwards. In the case of water and sanitation, we used the 2001 Census data in StatsSA’s SuperWeb database (StatsSA 2014) as it was not available in Development Indicators. We also used Census 2001 for household goods as it did not appear in the recent GHS’s. StatsSA’s historical revision of Labour Force Surveys (which preceded Quarterly Labour Force Surveys) (StatsSA 2009) was used for unemployment in 2001, as Development Indicators reported the narrow rather than the broad definition by province. For safety, we used the National Victims of Crime Survey 2003 (Burton et al. 2004). We could not find provincial data for three dimensions—housing, voice and income—as the indicators we used in the barometer were not reported.

## Results

### Environmental stress

**Fig. 2:**
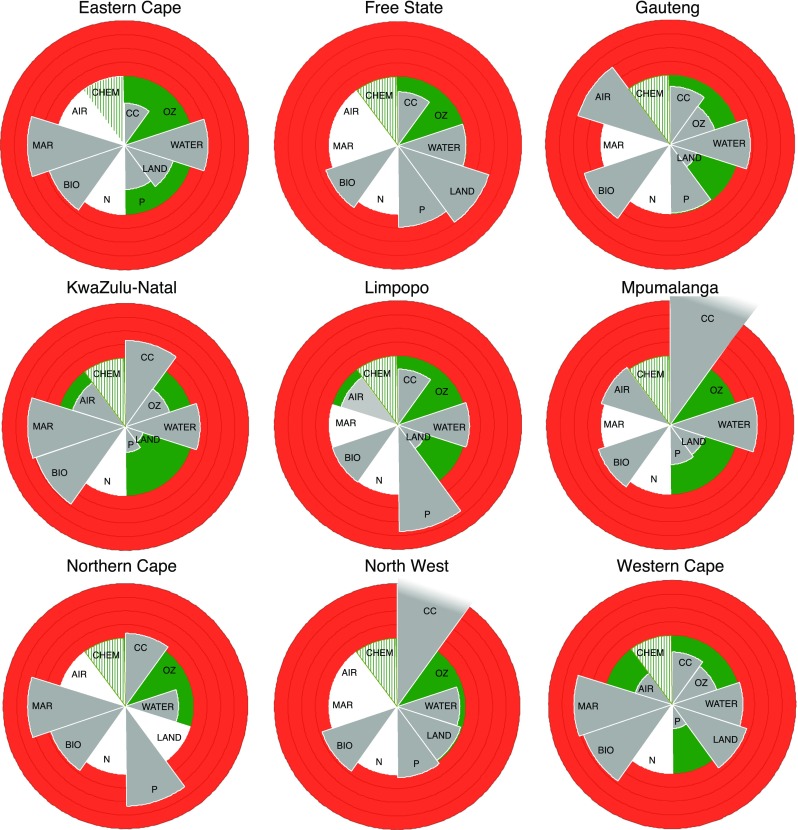
The nine provincial barometers for environmental stress in South Africa. *Grey wedges* plot the normalised status per dimension (see Table 4). Zero stress at the centre increasing to 100% at the boundary between the ‘safe environmental operating space’ (*green* area) and the unsafe environmental operating space (*red* area). White wedges indicate not relevant or no data available. *Striped green*/*white wedges* show the indicator was not defined. Dimensions are (clockwise from *top right*) climate change (CC), ozone depletion (OZ), freshwater use (WATER), arable land use (LAND), phosphorus loading (P), nitrogen cycle (N), biodiversity loss (BIO), marine harvesting (MAR), air pollution (AIR) and chemical pollution (CHEM)

**Fig. 3:**
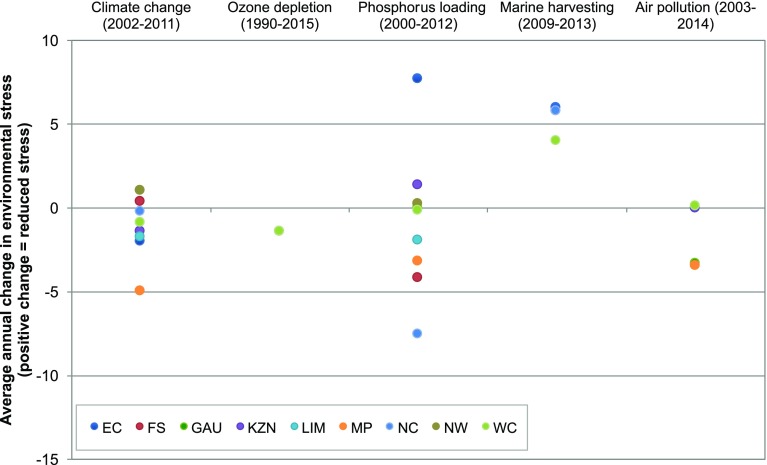
Average annual percentage change in environmental stress in the provinces. *Positive change* indicates decreased stress while *negative change* indicates increased stress. The time period varies for each dimension based on available data and is shown on the *x*-axis

The results for environmental stress are shown in Table 4 and Figs. 2 and 3. Table 4 provides the current status and boundary while the provincial barometers in Fig. 2 show the normalised status of each dimension, i.e. status as a percentage of the boundary. The barometers, therefore, show which provinces are exceeding their safe boundary and are contributing to the national boundary being exceeded. Figure 3 plots the trends, expressed as the annual change in the normalised status, for five of the environmental dimensions, as no comparable historical data was available for water use, arable land use or biodiversity loss due to a change in reporting methodologies. The trends in numbers are provided in SI Tables S4, S8, S12 and S13. The trends plot shows where the highest and lowest rate of change occurs among the provinces in the past 20 years.

**Table 4:** The current status (S), safe boundary (B) and normalised status (N) for dimensions of environmental stress for the provinces

Province	Climate change in 2011 (MtCO_2_)			Ozone depletion in 2015 (ODPt)			Freshwater use in 2011 (Mm^3^/a)			Arable land use in 2013 (ha)			Phosphorus loading in 2012 (mg/l)			Biodiversity loss in 2011 (%)			Marine harvesting in 2013 (%)			Air pollution in 2014 (µg/m^3^)		
	S	B	N	S	B	N	S	B	N	S	B	N	S	B	N	S	B	N	S	B	N	S	B	N
Eastern Cape	21	34	61	–	–	–	395	325	121	828,507	1,122,937	74	0.064	0.1	64	15	0	115	41	0	141	–	–	–
Free State	19	24	78	–	–	–	239	209	99	3,070,570	2,204,698	139	0.119	0.1	119	11	0	111	–	–	–	–	–	–
Gauteng	131	157	84	106	170	68	1,325	1,092	116	306,597	772,377	40	0.099	0.1	99	31	0	131	–	–	–	55.9	40	140
KwaZulu-Natal	90	71	126	19	30	68	736	566	109	691,483	2,537,768	27	0.037	0.1	37	38	0	138	43	0	143	32.3	40	81
Limpopo	26	32	81	–	–	–	258	207	104	967,732	2,296,820	42	0.154	0.1	154	1	0	101	–	–	–	34.8	40	87
Mpumalanga	75	32	237	–	–	–	248	183	127	1,278,717	2,393,780	53	0.057	0.1	57	10	0	110	–	–	–	41.9	40	105
Northern Cape	11	10	107	–	–	–	139	119	78	0	0	–	0.147	0.1	147	16	0	116	44	0	144	–	–	–
North West	56	29	189	–	–	–	211	179	92	1,631,535	1,661,912	98	0.104	0.1	104	16	0	116	–	–	–	–	–	–
Western Cape	49	64	76	83	133	68	529	443	103	876,367	767,777	103	0.035	0.1	35	36	0	136	43	0	143	22.5	40	56
South Africa	477	454	105	208	333	68	4,066	3,285	111	9,651,508	13,758,068	111	0.101	0.1	101	34	0	134	43	0	143	39.2	40	98
Mean (*N*)	115%			68%			105%			73%			91%			119%			143%			95%		
Range (*N*)	176%			0%			49%			112%			119%			37%			3%			84%		
SD (*N*)	56%			0%			14%			37%			42%			12%			1%			26%		
CV (*N*)	0.54			0.00			0.13			0.54			0.42			0.09			0.01			0.26		

Units given in brackets. See Table 2 for indicator descriptions. Normalised mean, range, standard deviation (SD) and coefficient of variation (CV) refer to the status as a percentage of the boundary

The results show that there is significant sub-national variation in environmental status and stress. The biggest variation across provinces occurs for climate change (range 176%—max. 237% min. 61%), phosphorus loading (range 119%—max. 154%, min. 35%) and air pollution (range 84%—max. 140%, min. 56%). The smallest range occurs for marine harvesting (3%), largely due to the overlap in geographic location of fisheries. Marine harvesting and biodiversity loss exceed the boundary in all relevant provinces, whereas ozone depletion is below the boundary in all provinces. Every province has between two and four dimensions that exceed the safe environmental boundary and need urgent attention. We find that for freshwater use, arable land use and air pollution, some of the provincial environmental boundaries have been exceeded despite their national boundary not being exceeded. KwaZulu-Natal has the highest risk of biodiversity loss (38% of ecosystems are endangered or critically endangered). Gauteng has the worst air pollution (PM10 concentration is 40% higher than the threshold). Mpumalanga has the highest carbon emissions intensity (137% over the GDP-based boundary) and water stress (demand is 27% higher than supply). The Free State has the most stressed arable land (39% of cultivated arable land is marginal). Phosphorus loading is highest in Limpopo (P levels are 54% above the acceptable threshold). Gauteng, KwaZulu-Natal and Western Cape contribute the most to total carbon emissions and ozone depletion.

Generally, there has been an increase in environmental stress across provinces over time, with the notable exception of marine harvesting. For the five dimensions that could be assessed, marine harvesting exhibits the most change nationally (17% decrease over a 4-year period) while CO_2_ emissions shows the least change (5% increase over an 9-year period). Phosphorus loading has the highest variation between provinces (range 15% per year). Overall environmental stress has been increasing fastest in Mpumalanga. The Eastern Cape and Mpumalanga have seen the most change and North West and Western Cape have seen the least change in the measured dimensions.

### Social deprivation

**Fig. 4:**
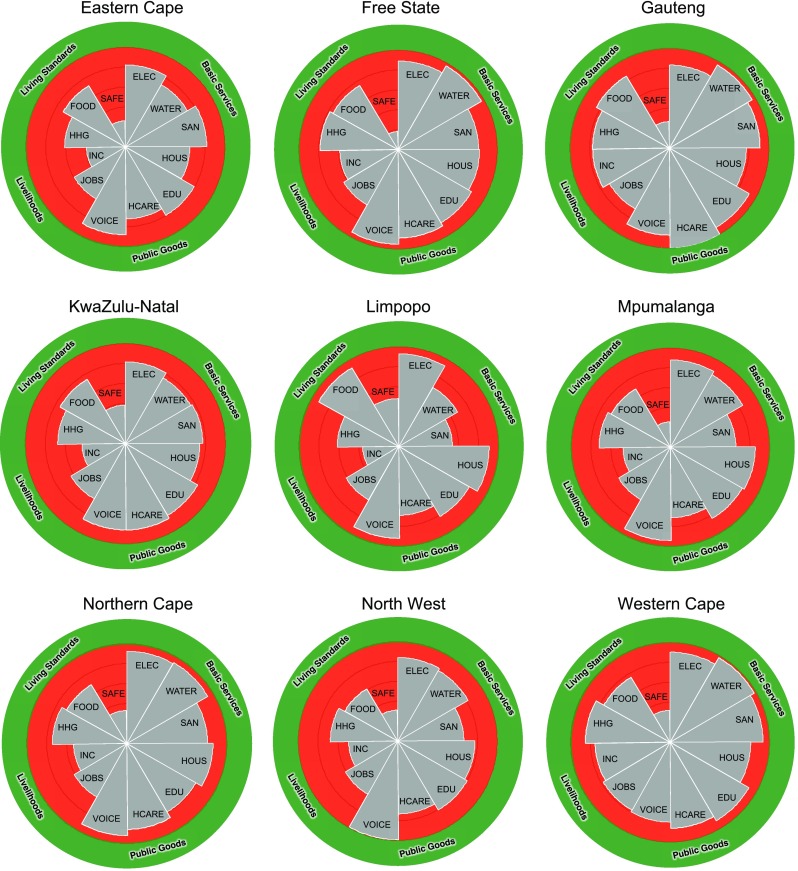
The nine provincial barometers for social deprivation in South Africa. *Grey wedges* plot the status per dimension (see Table 5). 100% deprivation at the centre decreasing to zero deprivation at the boundary between the ‘just social space’ (*green* area) and ‘unjust social space’ (*red* area). Dimensions are (clockwise from *top right*) electricity access (ELEC), water access (WATER), sanitation (SAN), housing (HOUS), education (EDU), health care (HCARE), voice (VOICE), jobs (JOBS), income (INC), household goods (HHG), food security (FOOD) and safety (SAFE)

**Fig. 5:**
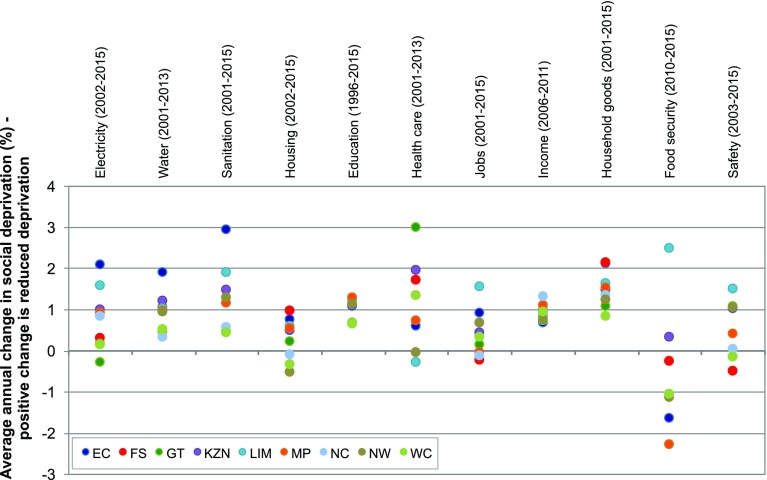
Average annual change in social deprivation in the provinces. Deprivation measured as percentage of population who are deprived. *Positive change* indicates reduced deprivation while *negative change* indicates increased deprivation. The time period varies for each dimension based on available data and is shown on the *x*-axis

The results for social deprivation are shown in Table 5 and Figs. 4 and 5. The status is expressed as a percentage for all dimensions and all boundaries (social floors) are zero, so no normalisation is necessary. Figure 5 shows the average annual change in percentage of the population who are deprived for eleven of the dimensions based on available data (no comparable historical data was available at provincial scale for voice). The actual numbers for the trends are provided in SI Table S15.

**Table 5:** The current status for dimensions of social deprivation for the provinces

Province	Basic services				Public goods			Livelihoods		Living standards		
	Electricity access (2015)	Water access (2013)	Sanitation (2015)	Formal Housing (2015)	Education (2015)	Health care (2014)	Voice (2011)	Jobs (2015)	Income (2011)	Household Goods (2015)	Food security (2015)	Safety (2015)
Eastern Cape	17.7	27.9	18.3	35.3	20.3	27.9	17.0	40.3	60.8	38.9	28.4	73.8
Free State	11.0	3.0	18.9	18.0	14.8	11.2	8.0	36.3	41.2	21.5	24.9	81.6
Gauteng	16.8	3.8	9.0	22.8	7.7	0.0	17.0	30.2	22.9	22.8	16.0	73.4
KwaZulu-Natal	178.3	24.5	24.3	25.6	17.0	13.9	17.0	36.8	56.6	32.4	25.3	61.6
Limpopo	7.1	31.7	46.2	9.5	18.8	31.2	14.0	38.6	63.8	38.3	8.2	51.9
Mpumalanga	12.2	15.8	34.2	14.6	16.8	29.1	15.0	39.4	52.1	28.2	31.7	74.5
Northern Cape	7.6	6.4	19.3	13.9	20.1	14.1	8.0	38.9	46.8	25.6	31.3	66.9
North West	16.0	18.3	33.6	22.5	19.8	26.9	5.0	38.9	50.5	32.3	39.0	68.8
Western Cape	9.8	2.3	6.7	19.0	8.8	13.9	24.0	22.0	24.7	15.0	24.0	68.0
South Africa	14.5	14.8	20.1	21.9	14.3	15.9	16.0	33.8	45.5	27.9	22.8	68.9
Range	11.2	29.4	39.5	25.8	13.0	31.2	19.0	18.3	40.9	23.9	30.8	29.7
SD	4.1	10.8	12.0	7.2	4.5	9.9	5.4	5.6	13.8	7.5	8.5	8.1
CV	0.28	0.73	0.60	0.33	0.32	0.63	0.45	0.17	0.30	0.27	0.37	0.12

Year of most recent data in brackets. All units are percentage population deprived. The social floor/boundary for all dimensions is 0%. See Table 3 for indicator descriptions

The provincial barometers show a similar pattern to the national barometer in that the most deprivation exists for safety, jobs and income while the least exists for water access. Some marked differences are evident, across multiple indicators, between provinces. The largest range is 41% for income and 40% for sanitation while the range for health care, food security, water access and safety are all close to 30%. These represent large variations in the living conditions of millions of people. In Limpopo 64% of the population lives below the poverty line and 46% of households do not have access to ventilated pit latrines or toilets. At the other end of the spectrum, in Gauteng only 23% live below the poverty line and only 9% do not have the minimum level of sanitation. Limpopo is the most deprived in water access, sanitation, health care and income. The Eastern Cape is the most deprived in formal housing, jobs and household goods. North West has the worst food security. Western Cape scores lowest on voice. KwaZulu-Natal has the lowest access to electricity. The Free State has the lowest levels of safety.

Overall, across nearly all provinces and social dimensions, there is a clear trend towards decreasing deprivation since 1994. Notable exceptions are food security in six provinces and safety, jobs and housing in three provinces. Limpopo, KwaZulu-Natal and Eastern Cape have seen the greatest annual decrease in deprivation, while the Western Cape has seen the lowest annual change in deprivation in the measured dimensions.

## Discussion

### Sub-national variation in sustainable development indicators

Our results show that South Africa is not on a sustainable development trajectory but there are promising signs that it could achieve this in the future. Social deprivation is decreasing across the country, and although environmental stress is increasing overall and in many provinces, there has been a reduction in stress from marine harvesting in all coastal provinces and CO_2_ emissions, phosphorus loading and air pollution (PM10) in some provinces. The province with fastest growing environmental stress, Mpumalanga, is experiencing a rapid increase in coal mining activity.

Social deprivation is decreasing faster in the historically disadvantaged provinces—particularly Limpopo, Eastern Cape and KwaZulu-Natal, which shows that government policy and programmes are working. However, this decrease is also partly due to migration to the cities and economic hubs, reducing the total population who need basic services and public goods in those provinces. StatsSA (2015b) estimate that for 2011–2016 over 100,000 people will have left the Eastern Cape and Limpopo provinces while net migration to Gauteng will be 543,109.

While our national barometer is a useful tool, the provincial barometers show that national reporting can hide significant heterogeneity in environmental status and social deprivation, including resource use, the means of obtaining income and a broader quality of life. The heterogeneity in the environmental status is a result of several factors such as, (a) the large natural variations in climate, terrain, soil and natural resources, (b) the varying population density and (c) varying economic activities (such as mining, manufacturing, farming and fishing) which have their own specific environmental impacts. The dimensions with the highest variation, namely climate change (CO_2_ emissions), phosphorus loading (total P concentrations) and air pollution (PM10 levels), all use indicators that measure pollutants and have strong social-ecological linkages. CO_2_ emissions and PM_10_ reflect different levels of industrialisation and/or electricity generation in different provinces while the main cause of phosphorus loading is the inadequate treatment of effluents discharged in river catchments (Oberholster and Ashton 2008). Ozone depletion has no provincial variation due to the calculation of the state and boundary both using an equal share approach.

The variation in social deprivation reflects the spatial inequality that was entrenched by the creation of ‘homelands’, partially self-governing territories set aside for black inhabitants of South Africa as part of the Apartheid agenda of racial segregation. These homelands had an extremely weak financial base and relied on transfer payments from the central South African government (Wittenberg 2006). Democracy in 1994 brought significant change to administrative boundaries in South Africa, with the nine provinces designed to combine homelands and parts of ‘white’ South Africa. Despite this, the Western Cape has no homeland areas while less than 3% of the area in Gauteng, Free State and Northern Cape were part of the homelands. These four provinces are the least socially deprived. In contrast, 34% of KwaZulu-Natal, 29% of the Eastern Cape, 27% of Limpopo and 25% of North West (DAFF 2015) were part of the homelands and they are still the most deprived today.

The variation in deprivation also reflects economic activity. The least deprived provinces, Gauteng and the Western Cape, are the first and third largest provincial economies, respectively. Gauteng’s GDP per capita is US$104,584, which is 16 times higher than the national average (StatsSA 2016d). There are also other factors that could affect the level of deprivation in the provinces, such as the quality of the provincial and municipal administration and the availability of skills. The provinces with the highest number of auditees with clean audit opinions in 2015–16 were the Western Cape (79%) and Gauteng (60%) (Auditor-General South Africa 2016).

### Barometers as policy tools

Barometers are becoming useful tools to support policy making in Africa and South Africa in particular. Examples include the Afrobarometer (Citizen Surveys 2013), the Reconciliation Barometer (Hofmeyr 2016), the Gauteng City–Region Observatory’s barometer of development (GCRO 2014) and the University of Western Cape’s social cohesion barometer (Struwig et al. 2011).

Our sustainable development barometer collates and summarises many of the key dimensions reported in the South African national reports on environmental and social indicators. Our provincial barometers are visual tools for decision-makers and communicate the range of key challenges that provincial governments face, including the current and past levels of risk. These data are seldom all presented at the provincial scale. Although social data are reported by province in household survey reports, they only appear in the online appendix of excel sheets in the annual Development Indicators reports (DPME 2015a). Environmental data are compiled at the national scale or by ecological unit (e.g. drainage basin or ecosystem type) by the national government (DEA 2012b, 2014a, 2016). While the provinces publish their own State of Environment reports, they have varying formats, indicators, frequency and data sources. Furthermore data availability varies considerably. For example, only four provinces have greenhouse gas inventories and each is calculated for a different year.

These differences make it difficult to compare provinces on an annual basis and track them over time. Our barometers and trend plots are novel in that they present comparable environmental and social data on key indicators over time for all provinces of South Africa in simple diagrams. Like the national indicator reports, they are user-friendly and accessible to a range of audiences. This includes decision-makers who need to make decisions on a broad spectrum of issues without necessarily being experts on most of those issues.

### Environmental governance and safe boundaries

There are two ways provincial data are used. The first is to monitor progress at the provincial level, and the second is to compare provinces at the national level. In this paper we have focused on the latter, and as a result some of the environmental indicators are not relevant for all provinces. This is because certain environmental stresses do not occur in that province (e.g. ozone depletion), are not relevant (e.g. marine harvesting in inland provinces), or do not meet the criteria of the national monitoring programme (e.g. air pollution). Only two provinces, KwaZulu-Natal and Western Cape, have data for all eight of the defined indicators. This clearly shows that the choice of national indicators should consider the application at sub-national scale if it is to be used to take action, and that in some cases (e.g. ozone depletion) a national indicator is necessary for international governance but is actionable only in some sub-national settings. It would be a useful exercise for each province to select their own set of dimensions and indicators using the decision flowchart developed in Cole et al. (2014). To maintain comparability, both sets of indicators (national and provincial) would be required.

The variation in environmental stress revealed by the disaggregation requires quite different responses from different provincial governments. The type of safe environmental boundary plays an important role in this. For Type A boundaries (based on international targets) the provinces must work together with the national government to determine their provincial boundary and action plans to meet international agreements. The methods we used for allocating responsibility would need to be debated and an acceptable approach agreed by all the relevant provinces to ensure that national targets are met. For ozone depletion, only three of the provinces (Gauteng, Western Cape and KwaZulu-Natal) would be involved, while all nine provinces would need to tackle climate change, although some provinces (particularly Mpumalanga, North West, KwaZulu-Natal and Gauteng) clearly have a larger role to play in reducing CO_2_ emissions. Although electricity is a national issue, efficiency in use might need a provincial role. Transport is a provincial and local government issue, especially in the big cities where specific choices about transport are needed. Agriculture and land use change emissions can be treated provincially as well.

For Type B boundaries (based on finite natural resources) the barometers show where there are opportunities and risks. Some provinces have unfarmed arable land or unutilised freshwater resources that could be developed and used to support economic growth and job creation. Other provinces are farming large areas on marginal arable land (e.g. Free State) or using more water than is ecologically sustainable (six out of nine provinces) and might require a strategic change in agricultural policy to avoid environmental degradation. It is impossible to redistribute arable land, and it may well be technically or economically unfeasible to transfer additional freshwater between the provinces. Therefore, the provincial barometers add a valuable insight into the nature of the water-food-energy nexus in the country, and can inform resource-dependent development strategies and Spatial Development Plans to achieve ‘spatial sustainability’ (DRDLR 2013).

For Type C boundaries (based on local biophysical thresholds), provinces need to identify local areas where the stress is occurring to take action. Local data already exist for phosphorus loading, biodiversity loss and marine harvesting and air pollution and the safe boundary is the same regardless of scale. The thresholds have been set by the national government and should be maintained locally to protect human health and the sustainability of jobs dependent on these natural resources. In the case of fertiliser use affecting the nitrogen cycle, both local status and safe boundaries need to be determined for different crops and farming regions.

### Defining social floors

The social indicators used in the barometer define national social floors, i.e. what is considered an unacceptable standard of living. These are largely based on data availability and reporting by national government (e.g. the national poverty line). They should ideally also have input from citizens, particularly those experiencing deprivation. South Africa’s National Development Plan (National Planning Commission 2012) aims to define the country’s minimum social floor and ensure that no one lives below this social floor by 2030. To begin to define a ‘democratic definition of poverty’ for South Africa, a module was included in the South African Social Attitudes Survey to obtain a nationally representative list of items, activities and services that the majority of people defined as ‘essential for everyone to have to enjoy an acceptable standard of living in South Africa today’ (Noble et al. 2013). The results of this survey and other participatory approaches could influence both national and sub-national indicators used in South African development reporting. It could also be used in the process of determining national SDG social indicators.

### SDG implementation

One of the chief aims of the current South African government is to reduce inequality. The SDGs and Agenda 2030 have committed to ‘leaving no-one behind’ and targets will not be considered as met unless they are met for the whole population. The spatial disaggregation of social deprivation that we have shown for South Africa’s nine provinces is an early case study of what is required for the national SDG implementation, and illustrates one approach to how that could be communicated. Our provincial barometers show that while the national statistics might show good progress overall there are some provinces that lag far behind.

While the SDGs call for disaggregation of social data, it is also useful to disaggregate environmental data so that it can be better monitored and managed. Both the state and boundary of each environmental indicator need to be disaggregated. In this paper, we have used three methods to disaggregate environmental data that could be used in any country across a range of indicators. The first method, “sharing a national total”, is a similar approach to that often used to share global commons by countries, for example in debates on climate change mitigation. While this approach is necessary when local data are not available, it is not ideal as sharing based on population, GDP or another metric will never be as accurate as aggregating local data. As it has become obvious in the climate change negotiations, it also leads to discussions on equity and historical responsibility that are hard to solve. The second method, “aggregating accessible local data”, is commonly used for national and global reporting. The challenge here is finding sufficient data that cover the whole country for annual or even for less frequent updates. We found that national reports often focus on the most stressed areas so not all local data are aggregated. While the ideal approach is to measure and collate large local data sets this is expensive. The SDSN estimates that a US$1 billion per annum will be required to enable 77 lower-income countries (40 of which are in Africa) to implement statistical systems capable of supporting and measuring the SDGs (SDSN 2015b). The third method, “finding the best fit between ecological units and administrative borders”, requires either estimation or expert knowledge and can be quite time consuming to be accurate. As many of the administrative borders within African countries do not follow natural terrain, ecological units will probably not match administrative regions. In South Africa, the CSIR has taken steps to overcome this challenge by demarcating ‘mesozones’—50 km^2^ units based on municipal boundaries, rivers, mountains, roads, population density and socio-economic character (Naude et al. 2007).

One big advantage of the SDGs for African countries is that each country chooses its own national indicators. While the global SDG indicators and other sustainability indices are useful guides, they may choose indicators that are not relevant to the national context. For example, the Sustainable Society Index (van de Kerk et al. 2014) uses SO_2_ emissions as a proxy for air pollution while the Environmental Performance Index (Hsu et al. 2016) uses PM_2.5_ and NO_2_. However, the South African government has identified PM_10_ as the main national concern. As our national and provincial barometers are tailored for South Africa (and informed by South African expert opinion) they could be used in the process of selecting the South African SDG indicators. However, as they do not cover all of the SDG targets they could merely be one tool in a much larger indicator selection process.

## Conclusion

As global environmental change and population growth strain natural resources in Africa, monitoring tools play an important part in helping countries solve their most pressing sustainability challenges. Our provincial barometers for inclusive sustainable development are visual tools for decision-makers that can communicate a range of key challenges that provincial governments face, including the current and past levels of risk. Our barometers and trend plots are novel in that they present comparable environmental and social data on key indicators over time for all South African provinces. They highlight the large variation in environmental stress and social deprivation across South Africa and emphasise the effect of geographical location on progress towards achieving sustainable development. In developing the barometers, we have highlighted three potential approaches to spatially disaggregate environmental data that could be used in other African countries for the SDG implementation. The study also provides insights into the ongoing debate on applying planetary boundaries at sub-global scales, particularly in developing countries.

## Electronic supplementary material

Below is the link to the electronic supplementary material.

Supplementary material 1 (DOCX 90 kb)

## References

[CR1] Auditor-General, South Africa (2016). Consolidated general report on the national and provincial audit outcomes 2015–16.

[CR2] Biggs R, Scholes RJ (2002). Land-cover changes in South Africa 1911–1993. S Afr J Sci.

[CR3] Boden TA, Marland G, Andres RJ (2011) Global, regional, and national fossil-fuel CO_2_ emissions. Carbon Dioxide Information Analysis Center (CDIAC), Oak Ridge National Laboratory, US Department of Energy, Oak Ridge, Tennessee

[CR4] Brentrup F, Palliere C (2010) Nitrogen use efficiency as an agro-environmental indicator. In: Workshop OECD (ed) Agri-environmental indicators: lessons learnt and future directions. Leysin, Switzerland

[CR5] Burton P, Du Plessis A, Leggett T (2004). National victims of crime survey South Africa 2003.

[CR6] Chamber of Mines of South Africa (2013) Putting South Africa first. Annual Report 2012/2013 Chamber of Mines of South Africa. Johannesburg

[CR7] Citizen Surveys (2013). Afrobarometer round 5 survey in South Africa summary of results.

[CR8] Cole MJ, Bailey RM, New MG (2014). Tracking sustainable development with a national barometer for South Africa using a downscaled “safe and just space” framework. Proc Natl Acad Sci USA.

[CR9] Collett A (2013) The impact of effective (geo-spatial) planning on the agricultural sector. In: South African Surveying and Geomatics Indaba 22–24 July 2013. Kempton Park, South Africa

[CR10] CSIR (2012) Workshop Report: Ocean Acidification and impacts on South African continental shelf ecosystems, 13–14 March 2012. Cape Town

[CR11] DAFF (2014). Status of the South African Marine Fishery resources 2014.

[CR12] DAFF (2015). Draft policy document on the preservation and development of agricultural land.

[CR13] Dao H, Pedizzu P, Chatenoux B (2015). Environmental limits and Swiss footprints based on planetary boundaries.

[CR14] de Vries W, Kros J, Kroeze C, Seitzinger SP (2013). Assessing planetary and regional nitrogen boundaries related to food security and adverse environmental impacts. Curr Opin Environ Sustain.

[CR15] DEA (2009) Government Notices No. 1210 National Environmental Management: Air Quality Act, 2004 National Ambient Air Quality Standards. Government Gazette No. 32816, Republic of South Africa

[CR16] DEA (2011) National list of ecosystems that are threatened and in need of protection. Government Gazette No. 34809, Republic of South Africa

[CR17] DEA (2012a) National waste information baseline report. draft. Department of Environmental Affairs, Government of South Africa. Pretoria

[CR18] DEA (2012b) 2012 South Africa environment outlook. Department of Environmental Affairs, Government of South Africa, Pretoria

[CR19] DEA (2013a) Long term adaptation scenarios Fagship research programme (LTAS) for South Africa. Summary for policy-makers. Department of Environmental Affairs, Government of South Africa, Pretoria

[CR20] DEA (2013b) Environmental sustainability indicators technical report 2011. Department of Environmental Affairs, Government of South Africa, Pretoria

[CR21] DEA (2013c) 2013 state of air summary. Department of Environmental Affairs, Government of South Africa. Cape Town

[CR22] DEA (2014a) Environmental sustainability indicators technical report 2012. Department of Environmental Affairs, Government of South Africa, Pretoria

[CR23] DEA (2014b) 2014 State of air report and National air quality indicator

[CR24] DEA (2015a) State of the oceans and coasts around South Africa 2014. Department of Environmental Affairs, Government of South Africa, Pretoria

[CR25] DEA (2015b) 2015 State of air report and national air quality indicator

[CR26] DEA (2016) Environmental indicators database. http://enviroindicator.environment.gov.za/cocoon/rsadb/docs/list. Accessed 3 Mar 2016

[CR27] Dearing JA, Wang R, Zhang K (2014). Safe and just operating spaces for regional social-ecological systems. Glob Environ Chang.

[CR28] DEAT (2006). South Africa environment outlook: a report on the state of the environment.

[CR29] DEAT (2009) Greenhouse gas inventory South Africa 1990 to 2000. Compilation under the United Nations Framework Convention on Climate Change (UNFCCC). National Inventory Report. p 89

[CR30] DPME (2013). Development indicators 2012.

[CR31] DPME (2015a) Development indicators 2014. Department of Planning, Monitoring and Evaluation, Government of South Africa, Pretoria

[CR32] DPME (2015b) Development indicators 2014 detailed excel sheets. Department of Planning, Monitoring and Evaluation, Government of South Africa, Pretoria

[CR33] DRDLR (2013) Spatial planning and land use management act, 2013. Republic of South Africa

[CR34] Driver A, Sink KJ, Nel JN (2012). National biodiversity assessment 2011: an assessment of South Africa’s biodiversity and ecosystems. Synthesis report.

[CR35] DWA (2013). National water resource strategy June 2013s Edition.

[CR36] DWA (2014) National eutrophication monitoring programme (NEMP) database. Department of Water Affairs, Government of South Africa

[CR37] DWAF (2004). National water resource strategy.

[CR38] European Comission (2014) Living well, within the limits of our planet: general union environment action programme to 2020. European Union, Luxembourg

[CR39] Fang K, Heijungs R, De Snoo GR (2015). Understanding the complementary linkages between environmental footprints and planetary boundaries in a footprint–boundary environmental sustainability assessment framework. Ecol Econ.

[CR40] FertASA (2013) Fertilizer consumption in South Africa 2012 (metric tonnes). Fertiliser Society of South Africa. http://www.fssa.org.za/Statistics/Estimated_fertilizer_use_by_crop_in_SA_for_2012.pdf.

[CR41] Gabrielsen P, Bosch P (2003) Environmental indicators: typology and use in reporting. EEA internal working paper

[CR42] Gasparatos A, Takeuchi K, Elmqvist T (2016). Sustainability science for meeting Africa’s challenges. Sustain Sci.

[CR43] GCRO (2014) Gauteng City-Region Observatory newsletter August 2014. 4(2):1–11.

[CR44] Hajer M, Nilsson M, Raworth K (2015). Beyond Cockpit-ism: four insights to enhance the transformative potential of the sustainable development goals. Sustainability.

[CR45] Hammond A, Adriaanse A, Rodenburg E, et al (1995) Environmental indicators: a systematic approach to measuring and reporting on environmental policy performance in the context of sustainable development

[CR46] Hofmeyr J (2016). SA reconciliation barometer 2015 political participation.

[CR47] Hsu A, Alexandre N, Cohen S (2016). 2016 environmental performance index.

[CR48] IAEG-SDG (2015) Meeting summary and work plan from the 2nd meeting of the IAEG-SDGs, 26–28 October 2015

[CR49] Kroll C (2015). Sustainable development goals: are rich countries ready?.

[CR50] McCafferty JR, Ellender BR, Weyl OLF, Britz PJ (2012). The use of water resources for inland fisheries in South Africa. Water SA.

[CR51] National Planning Commission (2012). National development plan 2030 our future—make it work.

[CR52] National Treasury (2015). Budget review 2015.

[CR53] Naude A, Badenhorst W, Zietsman L et al (2007) Geospatial Analysis platform—version 2: technical overview of the mesoframe methodology and South African Geospatial Analysis Platform. CSIR Report number: CSIR/BE/PSS/IR/2007/0104/B.

[CR54] NEDLAC (2012) Hydrochlorofluorocarbons (HCFC) Phase Out Plan for South Africa. NEDLAC Trade and Industry Chamber, Ferndale, South Africa

[CR55] Nel JL, Driver A (2012) South African National biodiversity assessment 2011: Technical Report. Vol 2: Freshwater component. CSIR Report Number CSIR/NRE/ECO/2012/0022/A. Stellenbosch

[CR56] Nicolai S, Hoy C, Berliner T, Aedy T (2015). Projecting progress: reaching the SDGs by 2030. ODI Flagship Report.

[CR57] Niedertscheider M, Gingrich S, Erb K-H (2012). Changes in land use in South Africa between 1961 and 2006: an integrated socio-ecological analysis based on the human appropriation of net primary production framework. Reg Environ Chang.

[CR58] Noble M, Barnes H, Wright G (2009). The South African Index of Multiple Deprivation 2001 at Datazone level.

[CR59] MWJ, Wright GC, Magasela WK, Ratcliffe A, Noble (2013). Developing a democratic definition of poverty in South Africa. J Poverty.

[CR60] Nykvist B, Persson Å, Moberg F (2013). National environmental performance on planetary boundaries: a study for the Swedish Environmental Protection Agency.

[CR61] Oberholster PJ, Ashton PJ (2008) State of the Nation Report: an overview of the current status of water quality and eutrophication in South African rivers and resevoirs: CSIR/NRE/WR/IR/2008/0075/C. Pretoria

[CR62] Palma JG (2011). Homogeneous middles vs. heterogeneous tails, and the end of the “Inverted-U”: it’s all about the share of the rich. Dev Change.

[CR63] Raworth K (2012) A safe and just space for humanity: Can we live within the doughnut? Oxfam Discussion Paper. Oxfam, Oxford, UK

[CR64] Republic of South Africa (2012) The constitution of the Republic of South Africa, 1996

[CR65] Rockström J, Steffen W, Noone K et al (2009a) A safe operating space for humanity. Nature 461:472–475. doi:10.1038/461472a 10.1038/461472a 19779433

[CR66] Rockström J, Steffen W, Noone K et al (2009b) Planetary boundaries: exploring the safe operating space for humanity. Ecol Soc 14:32

[CR67] Sachs J, Schmidt-Traub G, Kroll C et al (2016) SDG index and dashboards—global report. Bertelsmann stiftung and sustainable development solutions network (SDSN), New York

[CR68] Scenario Building Team (2007). Long term mitigation scenarios strategic options: technical summary.

[CR69] Schoeman JL, van der Walt M, Monnik KA et al (2002) Development and application of a land capability classification system for South Africa. Pretoria

[CR70] Schoeman F, Newby TS, Thompson MW, Van den Berg EC (2013). South African national land-cover change map. South Afr J Geomatics.

[CR71] SDSN (2015a) Indicators and a monitoring framework for sustainable development goals—launching a data revolution for the SDGs. Sustainable Development Solutions Network

[CR72] SDSN (2015b) Data for development: a needs assessment for SDG monitoring and statistical capacity development. Sustainable Development Solutions Network

[CR73] Sink K, Holness S, Harris L et al (2012) South African National biodiversity assessment 2011: Technical Report. Volume 4: Marine and coastal component. Pretoria

[CR74] StatsSA (2002) Statistical release P4141 generation and consumption of electricity December 2002. Statistics South Africa, Pretoria

[CR75] StatsSA (2009) Statistical release P0210 Labour Force Survey Historical Revision September Series 2000–2007. Statistics South Africa, Pretoria

[CR76] StatsSA (2012a) Statistical release P4141 Electricity generated and available for distribution January 2012. Statistics South Africa, Pretoria

[CR77] StatsSA (2012b) Statistical release P0441 Gross domestic product Annual estimates 2002–2011, Regional estimates 2002–2011, Third quarter 2012. Statistics South Africa, Pretoria

[CR78] StatsSA (2012c) Statistical release P0301.4 Census 2011. Statistics South Africa, Pretoria

[CR79] StatsSA (2014) SuperWeb database. In: Census 2011 Community Surv. database. http://interactive.statssa.gov.za/superweb/login.do. Accessed 25 Mar 2014

[CR80] StatsSA (2015a) Statistical release P0341 Victims of Crime Survey 2014/2015. Statistics South Africa, Pretoria

[CR81] StatsSA (2015b) Statistical release P0302 Mid-year population estimates 2015. Statistics South Africa, Pretoria

[CR82] StatsSA (2016a) Statistical release P0211 Quarterly Labour Force Survey Quarter 2: 2016. Statistics South Africa, Pretoria

[CR83] StatsSA (2016b) Statistical release P0318 General household survey 2015. Statistics South Africa, Pretoria

[CR84] StatsSA (2016c) Statistical release P0211 Quarterly Labour Force Survey Quarter 4: 2015. Statistics South Africa, Pretoria

[CR85] StatsSA (2016d) Statistical release P0441 Gross domestic product Fourth quarter 2015. Statistics South Africa, Pretoria

[CR86] Steffen W, Richardson K, Rockström J (2015). Planetary boundaries: guiding human development on a changing planet. Science.

[CR87] Struwig J, Davids YD, Roberts B et al (2011) Towards a social cohesion barometer for South Africa. Somerset West, South Africa

[CR88] UN General Assembly (2015). Transforming our world: The 2030 agenda for sustainable development.

[CR89] UNEP (2014) Megadiverse countries. In: Biodivers A-Z. http://www.biodiversitya-z.org/content/megadiverse-countries. Accessed 12 Aug 2016

[CR90] UNEP (2016) UNEP Ozone Secretariat Data Access Centre. http://ozone.unep.org/en/data-reporting/data-centre. Accessed 12 Aug 2016

[CR91] UNSD (2015a) Discussion paper on principles of using quantification to operationalize the SDGs and criteria for indicator selection

[CR92] UNSD (2015b) Millennium development goals indicators. http://mdgs.un.org/unsd/mdg/SeriesDetail.aspx?srid=749&crid=710. Accessed 26 Nov 2015

[CR93] van Ginkel C (2011). Eutrophication: present reality and future challenges for South Africa. Water SA.

[CR94] van Niekerk L, Turple JK (2012) South African National Biodiversity Assessment 2011: Technical Report. Volume 3: Estuary Component. CSIR Report Number CSIR/NRE/ECOS/ER/2011/0045/B. Stellenbosch

[CR95] van de Kerk G, Manuel A, Kleinjans R (2014). Sustainable society index SSI-2014.

[CR96] Wittenberg M, Bardhan P, Mookherjee D (2006). Decentralisation in South Africa. Decentralization and local governance in developing countries: a comparative perspective.

[CR97] World Bank (2016) Labor participation rate, total. In: World Bank Data. http://data.worldbank.org/indicator/SL.TLF.CACT.ZS/countries/1W?display=graph.

[CR98] Wright G, Noble M (2009). The South African index of multiple deprivation 2007 at municipality level.

