# Supplementary material for: Spatial variability in sustainable development trajectories in South Africa: provincial level safe and just operating spaces
Source: Sustain Sci. 2017 Feb 7;12(5):829–48. doi: 10.1007/s11625-016-0418-9 (PMC6086264; doi:10.1007/s11625-016-0418-9)
Supplement: Supplementary file 1 — Supplementary material 1 (DOCX 90 kb) [file 11625_2016_418_MOESM1_ESM.docx]

**Supplementary Information**

**Section A. Comparison of indicators in our barometer with the SDGs**

**Table S1. Comparison of our barometer, the SDGs and global indicators (SDSN 2015)**

| **Barometer** | | **Most relevant SDG goal and target** | | **Global indicator** |
| --- | --- | --- | --- | --- |
| **Dimension** | **Indicator** |  |  |  |
| Climate change | Annual direct CO_2_ emissions | 13.2 | Integrate climate change measures into national policies, strategies and plans | 62, 79 |
| Ozone depletion | Annual consumption of HCFC (ODt) | 12.4 | By 2020, achieve the environmentally sound management of chemicals and all wastes throughout their life cycle in accordance with agreed international frameworks… | 74 |
| Freshwater use | Annual consumption of freshwater (Mm^3^yr^-1^) | 6.4 | By 2030, substantially increase water-use efficiency across all sectors and ensure sustainable withdrawals of freshwater … | 49 |
| Land use change | Use of arable land for cropland (ha) | 2.4 | By 2030, ensure sustainable food production systems and implement sustainable and resilient agricultural practices… | 83 |
| Phosphorus cycle | Annual mean phosphorus concentration in reservoirs (mg/l) | 6.3 | By 2030 improve water quality by reducing pollution, eliminating dumping…halving the proportion of untreated wastewater… | 47 |
| Nitrogen cycle | Nitrogen application rate for maize production (kg/ha) | 2.4 | By 2030, ensure sustainable food production systems and implement sustainable and resilient agricultural practices… | 15 |
| Biodiversity loss | Endangered ecosystems (%) | 15.5 | Take urgent and significant action to reduce the degradation of natural habitats, halt the loss of biodiversity, and by 2020 protect and prevent the extinction of threatened species | 86 |
| Marine harvesting | Depleted fish stocks (%) | 14.4 | By 2020 effectively regulate harvesting and end overfishing, illegal, unreported and unregulated fishing practices and implement science-based management plans, in order to restore fish stocks in the shortest time feasible, at least to levels that can produce maximum sustainable yield | 82 |
| Air pollution | Average PM10 concentration (ug) | 3.9 | By 2030, substantially reduce the number of deaths and illnesses from air (indoor and outdoor) pollution | 69 |
| Chemical pollution | To be determined | 12.4 | By 2020, achieve the environmentally sound management of chemicals and all wastes throughout their life cycle in accordance with agreed international frameworks and significantly reduce their release to air, water and soil… |  |
| Energy access | Households without access to electricity | 7.1 | By 2030 ensure universal access to affordable, reliable and modern energy services | 51 |
| Water access | Households without access to water infrastructure >= RDP standard | 6.1 | By 2030 achieve universal and equitable access to safe and affordable drinking water for all | 45 |
| Sanitation | Households without access to at least ventilated improved pit latrines | 6.2 | By 2030 achieve access to adequate and equitable sanitation and hygiene for all, paying special attention to the needs of women and girls | 46 |
| Housing | Households not in formal dwellings | 11.1 | By 2030 ensure access for all to adequate, safe and affordable housing and basic services and upgrade slums |  |
| Education | Adults without more than 7 years of schooling (adult illiteracy rate) | 4.6 | By 2030 ensure all youth and significant proportion of adults, men and women, achieve literacy and numeracy | 33, 35 |
| Health care | Infant (<1 year) immunisation coverage | 3.8 | Achieve universal health coverage, including…access to quality essential health-care services and access to safe, effective, quality and affordable essential medicines and vaccines for all | 19 |
| Jobs | Broad unofficial unemployment rate (adults aged 15-64 available to work) | 8.5 | By 2030 achieve full and productive employment and decent work for all women and men, including young people and persons with disabilities, and equal pay for work of equal value | 57 |
| Income | Population living below the upper national poverty line | 1.2 | By 2030, reduce by half the proportion of people living below national poverty lines | 1 |
| Food | Households without adequate food | 2.1 | By 2030, end hunger and ensure that all people have access to adequate, safe and nutritious food all year round | 8 |
| Voice | Population who feel they can say what they think | 16.7 | Ensure responsive, inclusive, participatory and representative decision-making at all levels |  |
| Household goods | Households without a refrigerator |  |  |  |
| Safety | Households who feel unsafe walking alone in their area during the night | 16.1  5.2 | Significantly reduce all forms of violence and related death rates everywhere  By 2030 eliminate all forms of violence against women and girls | 94, 88 |

**Section B. Environmental Dimensions of the Barometer**

**Climate Change**

The provincial inventories: the Western Cape reported 41.3 MtCO_2_e energy emissions in 2009 (Western Cape Government 2013) – 13% of national energy consumption but 11% of national energy emissions, Gauteng reported 118.2 MtCO_2_ in 2007 (Tomaschek et al. 2012), the Eastern Cape reported 23.4 MtCO_2_ in 2008 (Government of the Eastern Cape 2011) and the Free State reported 22.6 MtCO_2_ in 2012 (YM Mashalaba & Associates 2012).

**Table S2. Disaggregating CO_2_ emissions to the provincial level using share of population (StatsSA 2012a), GDP (StatsSA 2014) and electricity (StatsSA 2016a) in 2011 (year of most recent available CO_2_ data)**

| **Province** | **Population share (%)** | **GDP share (%)** | **Electricity use share (%)** | **Status based on electricity use (MtCO_2_)** | **Boundary based on GDP (MtCO_2_)** | **Normalised status (proximity to boundary)** |
| --- | --- | --- | --- | --- | --- | --- |
| Eastern Cape | 12.7 | 7.5 | 4.4 | 20.8 | 34.0 | 61% |
| Free State | 5.3 | 5.3 | 4.0 | 18.9 | 24.0 | 78% |
| Gauteng | 23.7 | 34.5 | 27.5 | 131.3 | 156.5 | 84% |
| KwaZulu-Natal | 19.8 | 15.7 | 18.8 | 89.8 | 71.2 | 126% |
| Limpopo | 10.4 | 7.1 | 5.4 | 26.0 | 32.2 | 81% |
| Mpumalanga | 7.8 | 7.0 | 15.8 | 75.3 | 31.8 | 237% |
| Northern Cape | 2.2 | 2.2 | 2.2 | 10.7 | 10.0 | 107% |
| North West | 6.8 | 6.5 | 11.7 | 55.7 | 29.5 | 189% |
| Western Cape | 11.2 | 14.2 | 10.2 | 48.8 | 64.4 | 76% |
| *South Africa* | *100* | *100* | *100* | *477.2* | *453.7* | *105%* |

**Table S3. Downscaling climate change to the provincial level using share of GDP (StatsSA 2012b) and electricity (StatsSA 2016a) in 2002 (year of oldest available electricity use data)**

| **Province** | **GDP (%)** | **Estimated boundary (MtCO_2_)** | **Electricity use share (%)** | **Estimated status (MtCO_2_)** | **Proximity to Boundary** | **Change from 2002 to 2011** |
| --- | --- | --- | --- | --- | --- | --- |
| Eastern Cape | 7.8 | 27.1 | 3.4 | 11.8 | 44% | +17% |
| Free State | 5.6 | 19.5 | 4.6 | 16.0 | 82% | -4% |
| Gauteng | 33.5 | 116.5 | 25.6 | 89.0 | 76% | +8% |
| KwaZulu-Natal | 16.3 | 56.7 | 18.5 | 64.3 | 113% | +13% |
| Limpopo | 6.7 | 23.3 | 4.2 | 14.6 | 63% | +18% |
| Mpumalanga | 7.1 | 24.7 | 12.5 | 43.5 | 176% | +61% |
| Northern Cape | 2.2 | 7.6 | 2.1 | 7.3 | 95% | -12% |
| North West | 6.6 | 22.9 | 13.1 | 45.5 | 198% | -9% |
| Western Cape | 14.1 | 49.0 | 9.7 | 33.7 | 69% | +7% |
| *South Africa* | *100* | *347.7* | *100* | *347.7* | *100%* | *+5%* |

**Ozone Depletion**

**Table S4. Estimation of consumption of HCFCs by province**

|  | **Actual provincial consumption in 2010** | | | | | **Estimated provincial consumption in 2015** | |
| --- | --- | --- | --- | --- | --- | --- | --- |
| **Province** | **HCFC-141b in million tonnes*** | **% HCFC-141b** | **HCFC-22 in million tonnes**** | **% HCFC-22** | **% Total HCFC** | **Status in ODPt** | **Boundary in ODPt** |
| Eastern Cape | 0.6 | 0 | 0.0 | 0 | 0 | - | - |
| Free State | 0.0 | 0 | 0.0 | 0 | 0 | - | - |
| Gauteng | 1,391.7 | 77 | 958.0 | 26 | 51 | 106.2 | 169.7 |
| KwaZulu-Natal | 344.2 | 19 | 0.0 | 0 | 9 | 18.7 | 29.9 |
| Limpopo | 0.0 | 0 | 0.0 | 0 | 0 | - | - |
| Mpumalanga | 0.0 | 0 | 0.0 | 0 | 0 | - | - |
| Northern Cape | 0.0 | 0 | 0.0 | 0 | 0 | - | - |
| North West | 0.0 | 0 | 0.0 | 0 | 0 | - | - |
| Western Cape | 66.9 | 4 | 2,681.0 | 74 | 40 | 83.3 | 133.1 |
| *South Africa* | *1,803.4* | *100* | *3,639.0* | *100* | *100* | *208.3^* | *332.7* |

* Based on the foam sector baseline data in the HCFCs Phase Out Plan (NEDLAC 2012)

** Based on the largest importers in 2009/10 in the HCFCs Phase Out Plan

^ Reported by the United Nations Environment Program Ozone Secretariat (UNEP 2016)

**Freshwater Use**

Table S5 shows the total supply, demand (including transfers) and ecological reserve (as percentage of mean annual runoff, MAR) for each province for the year 2000 using the sub-areas data in the first National Water Resource Strategy, NWRS (DWAF 2004). The only major overlap was in two sub-areas of the Lower Vaal WMA, shared between the North West and Northern Cape; here we split the supply and demand equally between the two provinces. This data was not updated in the 2013 NWRS. Although two of the provincial SOE reports quote supply and demand values we did not use them as we did not know how they would affect the other provinces (Western Cape: supply 2522 Mm^3^/a, demand 2637 Mm^3^/a; Eastern Cape: supply 2181 Mm^3^/a, demand 1654 Mm^3^/a).

From 2004 to 2013 the Department of Water Affairs and Sanitation (DWS) undertook a large programme of work to develop water reconciliation strategies for the metropolitan areas (metros) as well as all towns and villages in South Africa to inform water resource investment and management decisions (DWA 2013). The eight metros are addressed in strategies for the major water supply systems (WSS): Western Cape WSS (DWAF 2007), Algoa WSS (DWA 2011), Amatole WSS (DWAF 2011), Greater Bloemfontein WSS (DWA 2012), Vaal WSS (DWAF 2009) and KwaZulu-Natal coast and metros WSS (DWA 2009). We used 2011 data for all metros. In the DWS’s ‘All Town Studies’ towns, villages and settlements were grouped into 838 logical clusters based on their water supply. The All Town Studies reported current (2007/8) and projected (2010 to 2035) water requirements (demand) and yields (supply). We aggregated the data in these studies to the provincial level shown in Table S6. The final column shows the supply minus the ecological reserve, calculated using the reserve as a percentage of MAR.

**Table S5. Status and boundaries for Freshwater Use in 2000 and 2011 by province**

|  | **National Water Resource Strategy (DWAF 2004) 2000** | | | **Reconciliation Strategies (DWS)**  **2008-2011** | | |
| --- | --- | --- | --- | --- | --- | --- |
| **Province** | **Demand (Mm^3^/a)** | **Sustainable supply (Mm^3^/a)** | **Reserve as % MAR** | **Demand (Mm^3^/a)** | **Supply (Mm^3^/a)** | **Supply less Reserve (Mm^3^/a)** |
| Eastern Cape | 1,378 | 1,896 | 14.7 | 395 | 382 | 325 |
| Free State | 870 | 918 | 12.5 | 207 | 239 | 209 |
| Gauteng | 1,605 | 1,649 | 17.6 | 1,268 | 1,325 | 1,092 |
| KwaZulu-Natal | 1,779 | 1,735 | 23.1 | 617 | 736 | 566 |
| Limpopo | 1,382 | 1,166 | 19.7 | 214 | 258 | 207 |
| Mpumalanga | 1,472 | 1,214 | 26.1 | 233 | 248 | 183 |
| Northern Cape | 1,661 | 2,032 | 14.5 | 93 | 139 | 119 |
| North West | 571 | 570 | 15.0 | 165 | 211 | 179 |
| Western Cape | 2,025 | 1,936 | 16.2 | 456 | 529 | 443 |
| *South Africa* | *12,743* | *13,116* | *19.2* | *3,648* | *4,066* | *3,285* |

**Arable Land Use**

The national land capability classification defines eight Classes. Classes I, II and III comprise rain-fed arable land of acceptable quality while Class IV is marginal arable land. The other four classes are non-arable.

**Table S6. Land capability and cultivated land use by province (Data sources: (DAFF 2015))**

| **Province** | **Total Land Area (ha)** | **Land Capability Class I-III Area (ha)** | **Status: Cultivated Class I-IV Land (ha)** | **Boundary: Available Land Capability Class I-III Area (ha)*** | **Cultivated Class I-IV Land as % of Available Class I-III Land** |
| --- | --- | --- | --- | --- | --- |
| Eastern Cape | 16,882,162 | 1,273,249 | 828,507 | 1,122,937 | 74% |
| Free State | 12,980,148 | 2,254,177 | 3,070,570 | 2,204,698 | 139% |
| Gauteng | 1,817,148 | 1,093,904 | 306,597 | 772,377 | 40% |
| KwaZulu-Natal | 9,322,929 | 3,097,604 | 691,483 | 2,537,768 | 27% |
| Limpopo | 12,573,636 | 2,534,914 | 967,732 | 2,296,820 | 42% |
| Mpumalanga | 7,646,428 | 2,957,734 | 1,278,717 | 2,393,780 | 53% |
| Northern Cape | 37,279,987 | 0 | 0 | 0 | - |
| North West | 10,486,312 | 1,777,280 | 1,631,535 | 1,661,912 | 98% |
| Western Cape | 12,935,453 | 895,807 | 876,367 | 767,777 | 114% |
| *South Africa* | *121,924,881* | *15,884,669* | *9,651,508* | *13,758,068* | *70%* |

* Original land capability area less land that has been permanently transformed, or is used for forestry plantations or is formally protected for biodiversity conservation

**Phosphorus Loading**

**Table S7. Phosphorus loading by province**

| **Province** | **Drainage basins** | **Status: Total Phosphorus in reservoirs (mg/l)** | | **Boundary: Total Phosphorus in reservoirs (mg/l)** | **Annual change**  **2000 to 2012 (%)** |
| --- | --- | --- | --- | --- | --- |
|  |  | **2012** | **2000** |  |  |
| Eastern Cape | P, R, S, T | 0.064 | 0.156 | 0.1 | -7.7 |
| Free State | C, D | 0.119 | 0.069 | 0.1 | 4.2 |
| Gauteng | A, B, C | 0.099 | 0.099 | 0.1 | 0.0 |
| KwaZulu-Natal | U, V, W | 0.037 | 0.053 | 0.1 | -1.3 |
| Limpopo | A, B | 0.154 | 0.131 | 0.1 | 1.9 |
| Mpumalanga | B, X | 0.057 | 0.019 | 0.1 | 3.2 |
| Northern Cape | D | 0.147 | 0.056 | 0.1 | 7.6 |
| North West | A, C | 0.104 | 0.107 | 0.1 | -0.3 |
| Western Cape | E, F, H, K | 0.035 | 0.033 | 0.1 | 0.2 |
| *South Africa* | *ALL* | *0.101* | *0.074* | *0.1* | *2.3* |

**Nitrogen** **Cycle**

**Table S8. Total maize production and estimated fertiliser use by province in 1990/91 and 2012/13 (Source data: Grain SA 2013 and FSSA 2013)**

| **Province** | **Status in 1990/91** | | | **Status in 2012/13** | | | **Boundary (kgN per ha)** |
| --- | --- | --- | --- | --- | --- | --- | --- |
|  | **'000 ha planted** | **tN** | **kgN per ha** | **'000 ha planted** | **tN** | **kgN per ha** |  |
| Western Cape | 2 | 137 | 69 | 3.3 | 272 | 82 | 144 |
| Northern Cape | 19 | 1302 | 69 | 53.2 | 4 388 | 82 | 144 |
| Free State | 929 | 63644 | 69 | 1 230.0 | 101 462 | 82 | 144 |
| Eastern Cape | 33 | 2261 | 69 | 18.7 | 1 543 | 82 | 144 |
| Kwazulu-Natal | 82 | 5618 | 69 | 95.0 | 7 837 | 82 | 144 |
| Mpumalanga | 591 | 40488 | 69 | 470.0 | 38 770 | 82 | 144 |
| Limpopo | 43 | 2946 | 69 | 53.5 | 4 413 | 82 | 144 |
| Gauteng | 134 | 9180 | 69 | 117.5 | 9 693 | 82 | 144 |
| North West | 1374 | 94129 | 69 | 740.0 | 61 042 | 82 | 144 |
| *South Africa* | *3207* | *291 704* | *69* | *2 781.2* | *229 420* | *82* | 144 |

**Biodiversity Loss**

In the NBA estuary technical report (van Niekerk and Turple 2012), (Table 10.5 on pages 119-120) threat status is reported by administrative district, hence we could calculate the area and number of EN and CR estuarine ecosystems for four provinces, shown in Table S8 and S9. In the marine and coastal technical report (Sink et al. 2012), threat status is reported for 58 coastal, 62 offshore benthic and 16 offshore pelagic habitat types (Table 14 pages 162-165). These can be grouped into 11 geographic regions – Agulhas, Delagoa, Namaqua, Natal, Natal-Delagoa, South Atlantic, Southeast Atlantic, Southern Benguela, Southwest Indian, Southwestern Cape and Pelagic. As the provinces do not have jurisdiction over the offshore regions, we focused on coastal and inshore habitat types only. We matched the regions to the four coastal provinces to calculate the area and number of EN and CR coastal and inshore habitat types (see Table S10 and S11). There is some overlap between the provinces due to the large geographical spread of the regions so in some cases a region was assigned to two provinces.

We could not determine the threat status for freshwater ecosystems (rivers and wetlands) from the NBA 2011, as they are downscaled to the Water Management Area level (Nel and Driver 2012), which does not match well to the provinces.

The threat status of 567 terrestrial ecosystems was based on the National List of Ecosystems that are Threatened and Need Protection (DEA 2011) which reports the original area and remaining natural area for 53 CR ecosystems and 64 EN ecosystems, and records the province. The remaining area is reported as a percentage of the original area, which gives a measure of extinction or habitat loss only, rather than threat status. The remaining area is also reported as a percentage of the area of the province, however we wanted to use the original area to determine the total endangered (CR and EN) terrestrial ecosystems as this will then incorporate the habitat loss i.e. both the area at risk of crossing the safe boundary and the area that has already crossed the safe boundary. We used the original CR+EN areas as a percentage of total province area, calculated by SANBI for the NBA 2011, shown in Table S10. The total number of habitats is not reported so we could not calculate percentages per province.

There are two options for determining a single provincial value for biodiversity loss – we can either measure it based on habitat area (Table S10) or based on number of ecosystems, estuaries and habitat types (Table S11). Due to the lack of data for the latter, and advice from experts at SANBI, we averaged the ecosystems by area to obtain a single value (% CR+EN) per province. We kept the safe provincial boundary the same as the national boundary i.e. no ecosystems should be endangered or critically endangered.

For the trends analysis, we reviewed the 2004 National Spatial Biodiversity Assessment (NSBA) (Driver et al. 2005), which was the first time South Africa calculated ecosystem threat status rather than species threat status. Estuarine ecosystems were not assigned threat status and the marine and coastal ecosystems were not assessed by habitat area so neither could be determined per province. Although threat status per terrestrial vegetation type was reported, this was not done by province. In addition, the thresholds for EN and VU ecosystems were much higher in the NBSA 2004 than the NBA 2011; 60% of original extent rather than +15% for EN and 80% rather than 60% for VU. It was changed for NBA 2011 as it was unworkable as CR and EN ecosystems trigger Environmental Impact Assessments, and was not as defensible from a science point of view (Driver et al. 2005). This means that the two assessments are not comparable and we were not able to plot the change in biodiversity loss.

**Table S9. Calculation of provincial critically endangered (CR) and endangered (EN) ecosystems using habitat area**

| **Province** | **Terrestrial ecosystems** | | **Estuarine ecosystems** | | **Coastal and inshore ecosystems** | | **Total** |
| --- | --- | --- | --- | --- | --- | --- | --- |
|  | **Original area (ha) CR+EN** | **%Total Area CR+EN** | **Area (ha) CR+EN** | **%Total Area CR+EN** | **Area (km^2^) CR+EN** | **%Total Area CR+EN** | **Average % CR+EN** |
| Eastern Cape | 106 312 | 0.6 | 3 525 | 41 | 110 | 2 | 14 |
| Free State | 1 466 064 | 11.3 | - | - | - | - | 11 |
| Gauteng | 557 695 | 30.7 | - | - | - | - | 31 |
| KwaZulu-Natal | 853 368 | 9.0 | 58 261 | 93 | 301 | 13 | 34 |
| Limpopo | 165 822 | 1.3 | - | - | - | - | 1 |
| Mpumalanga | 784 910 | 10.3 | - | - | - | - | 10 |
| Northern Cape | 75 147 | 0.2 | 243 | 19 | 2768 | 28 | 9 |
| North West | 1 672 581 | 15.9 | - | - | - | - | 16 |
| Western Cape | 2 181 136 | 16.8 | 14 817 | 80 | 1286 | 10 | 34 |
| *South Africa* | *7 863 036* | *6.4* | *73 947* | *81* | *4464* | *15* | *29* |

**Table S10. Calculation of provincial critically endangered (CR) and endangered (EN) ecosystems using number of estuaries for estuarine ecosystems and habitat types for inshore and coastal ecosystems**

| **Province** | **Estuarine ecosystems** | | **Inshore and coastal ecosystems** | |
| --- | --- | --- | --- | --- |
|  | **No. Estuaries CR+EN** | **% Estuaries CR+EN** | **Habitat Types CR+EN** | **% Habitat Types CR+EN** |
| Eastern Cape | 13 | 8 | 3 | 20 |
| Free State | - | - | - | - |
| Gauteng | - | - | - | - |
| KwaZulu-Natal | 21 | 28 | 3 | 9 |
| Limpopo | - | - | - | - |
| Mpumalanga | - | - | - | - |
| Northern Cape | 2 | 50 | 14 | 48 |
| North West | - | - | - | - |
| Western Cape | 27 | 51 | 10 | 33 |
| *South Africa* | *63* | *22* | *30* | *38* |

The updated planetary boundaries use the Biodiversity Intactness Index (BII), a measure of the ‘average abundance of wild population relative to their reference populations’ (Biggs et al. 2006), for regional boundaries for biodiversity integrity. Biggs et al (2006) calculated the BII for South Africa in 1995 to be 81% i.e. terrestrial plants and vertebrates populations have declined by 19% from their presumed pre-colonial levels. At a provincial level, the BII ranged from 64% for Gauteng to 92% for the Northern Cape. As the BII is scale-independent and has already been used at national, provincial and local scale in South Africa makes it an attractive indicator for the barometer. It does not include marine and freshwater ecosystems, however, and has not been updated since 2006, thus the data are 20 years out of date. This means it does not meet our selection criteria.

**Marine harvesting**

Table S11 shows the number of species and depleted species reported in the Status of the South African Marine Fishery Resources for the years 2009 (DAFF 2010) and 2013 (DAFF 2014). The fisheries are abalone, Agulhas sole, Cape hakes, Cape horse mackerel, linefish, netfish, oysters, Patagonian toothfish, prawns, seaweeds, sharks, small pelagic fish, South coast rock lobster, squid, tunas and swordfish, West coast rock lobster, and White mussels and small invertebrates.

**Table S11. Marine harvesting in 2013, 2011 and 2009 by province**

| **Province** | **Number of fisheries** | **2013** | | **2009** | |
| --- | --- | --- | --- | --- | --- |
|  |  | **Number of species** | **Depleted species** | **Number of species** | **Depleted species** |
| Eastern Cape | 15 | 39 | 14 (41%) | 49 | 26 (65%) |
| KwaZulu-Natal | 8 | 32 | 12 (43%) | 44 | 25 (66%) |
| Northern Cape | 12 | 34 | 14 (44%) | 45 | 25 (66%) |
| Western Cape | 16 | 45 | 17 (43%) | 53 | 27 (60%) |
| *South Africa* | *17* | *49* | *18 (43%)* | *54* | *27 (60%)* |

**Air Pollution**

For some air quality monitoring stations, monitoring started in 1994 while for others it only started in 2013. Other points have only one or two readings over that period. So it is difficult to identify a starting point to assess the provinces. To plot the trend, we decided to use the years 2003 and 2004 to ensure all relevant provinces were covered. This did mean that we only had 22 data points in total for 2000 whereas we had 50 data points in 2014. The North West province has only one monitoring station in Rustenburg and only has measurements reported for 2003 and 2004. We used the same boundary of 40 ug/m^3^ for 2000 and 2014 in our trend analysis.

**Table S12. Air quality (PM10 concentration in ug/m^3^) by province**

| **Province** | **Monitoring point locations** | **2014** | | **2003/04** | | **Annual change 2004 to 2014** |
| --- | --- | --- | --- | --- | --- | --- |
|  |  | **Status** | **Boundary** | **Status** | **Boundary** |  |
| Gauteng | Tshwane (6), Vaal Triangle Priority Area (6), Eskom (1) | 55.9 | 50 | 51.5 | 40 | 3.7 |
| KwaZulu-Natal | eThekwini (4), Richards Bay (4) | 32.3 | 50 | 40.2 | 40 | 0.0 |
| Limpopo | Waterberg-Bonjanala Priority Area (3) | 34.8 | 50 | - | - | - |
| Mpumalanga | Eskom (9), Highveld Priority Area (5) | 41.9 | 50 | 33.3 | 40 | 3.8 |
| North West | Rustenburg (0) | - | - | 62.9 | 40 | - |
| Western Cape | Cape Town (7), towns (5) | 22.5 | 50 | 28.6 | 40 | -0.1 |
| *South Africa* | *(41)* | *39.2* | *50* | *39.8* | *40* | *1.8* |

**Section C. Social Dimensions of the Barometer**

**Table S13. National and provincial results for the South Africa Afrobarometer Round 5 Study in 2011**

| **Question** | **Response** | **EC** | **FS** | **GAU** | **KZN** | **LIM** | **MP** | **NC** | **NW** | **WC** | **SA** | **Range** |
| --- | --- | --- | --- | --- | --- | --- | --- | --- | --- | --- | --- | --- |
| How free are you to say what you think? | Not free + Not very free | 17 | 8 | 17 | 17 | 14 | 15 | 8 | 5 | 24 | 16 | 19 |
| How free are you to join any political organisation? | Not free + Not very free | 12 | 5 | 13 | 14 | 8 | 6 | 8 | 1 | 20 | 12 | 19 |
| How free are you to choose who to vote for without feeling pressured? | Not free + Not very free | 9 | 5 | 9 | 11 | 8 | 3 | 4 | 2 | 15 | 9 | 13 |
| Have you attended a community meeting in the past year? | No, would never +  No, would if had the chance | 45 | 43 | 37 | 43 | 31 | 31 | 43 | 45 | 62 | 42 | 31 |
| Have you got together with others to raise an issue in the past year? | No, would never +  No, would if had the chance | 59 | 54 | 47 | 65 | 45 | 47 | 57 | 72 | 70 | 57 | 27 |
| Have you contacted a government department to raise an issue in the past year? | No, would never +  No, would if had the chance | 91 | 79 | 83 | 83 | 74 | 77 | 81 | 91 | 85 | 83 | 17 |
| Have you contacted radio, TV or newspaper to complain about an issue in the past year? | No, would never +  No, would if had the chance | 98 | 95 | 90 | 90 | 86 | 90 | 97 | 94 | 93 | 92 | 12 |
| Have you attended a demonstration or protest march in the past year? | No, would never +  No, would if had the chance | 93 | 83 | 87 | 89 | 75 | 89 | 86 | 89 | 90 | 87 | 18 |
| Do you rate the 2009 national election as free and fair? | Not free and fair | 4 | 3 | 2 | 3 | 4 | 2 | 5 | 1 | 5 | 3 | 4 |
| How much of a democracy is SA today? | Not a democracy | 4 | 3 | 1 | 3 | 4 | 0 | 4 | 1 | 6 | 3 | 6 |
| During election campaigns, do you fear becoming a victim of political intimidation or violence? | A lot + Somewhat | 19 | 11 | 17 | 33 | 18 | 19 | 11 | 26 | 20 | 21 | 22 |
| How much do you trust your local councillor? | Not at all + Just a little | 75 | 50 | 57 | 52 | 53 | 51 | 37 | 54 | 45 | 56 | 38 |
| How many local councillors are involved in corruption? | All of them + Most of them | 45 | 57 | 53 | 64 | 43 | 51 | 34 | 69 | 34 | 51 | 35 |
| How much do local councillors listen to what people like you have to say? | Never | 67 | 44 | 34 | 38 | 29 | 46 | 30 | 31 | 41 | 37 | 38 |

**Table S14. Annual average change in social deprivation (%)**

| **Province** | **Basic services** | | | | **Public goods** | | **Livelihoods** | | **Living standards** | | |
| --- | --- | --- | --- | --- | --- | --- | --- | --- | --- | --- | --- |
|  | **Energy (2002-2015)** | **Water (2001-2013)** | **Sanitation (2001-2015)** | **Housing (2002-2015)** | **Education (1996-2015)** | **Health Care (2001-2013)** | **Jobs (2001-2015)** | **Income (2006-2011)** | **Household Goods (2001-2015)** | **Food Security (2010-2015)** | **Safety (2003-2015)** |
| Eastern Cape | 2.1 | -1.9 | 2.9 | 0.7 | 1.1 | -0.6 | 0.9 | -0.7 | 2.1 | -1.6 | -0.2 |
| Free State | 0.3 | -1.0 | 1.9 | 1.0 | 1.1 | -1.7 | -0.3 | -0.9 | 2.1 | -0.3 | -0.5 |
| Gauteng | -0.3 | -0.5 | 0.5 | 0.2 | 0.7 | -3.0 | 0.1 | -0.7 | 1.1 | 0.3 | 1.1 |
| KwaZulu-Natal | 1.0 | -1.2 | 1.5 | 0.5 | 1.1 | -2.0 | 0.4 | -1.0 | 1.5 | 0.3 | 1.0 |
| Limpopo | 1.6 | -1.0 | 1.9 | 0.6 | 1.2 | 0.3 | 1.5 | -0.8 | 1.6 | 2.5 | 1.5 |
| Mpumalanga | 0.9 | -1.0 | 1.2 | 0.5 | 1.3 | -0.7 | -0.1 | -1.1 | 1.5 | -2.3 | 0.4 |
| Northern Cape | 0.8 | -0.4 | 0.6 | -0.1 | 1.1 | -1.3 | -0.1 | -1.3 | 1.3 | -1.1 | 0.0 |
| North West | 0.2 | -0.9 | 1.3 | -0.5 | 1.1 | 0.0 | 0.7 | -0.7 | 1.2 | -1.1 | 1.0 |
| Western Cape | 0.1 | -0.5 | 0.4 | -0.3 | 0.6 | -1.3 | 0.3 | -0.9 | 0.8 | -1.1 | -0.2 |
| *South Africa* | *0.6* | *-1.0* | *1.4* | *0.3* | *1.0* | *-0.4* | *0.4* | *-0.9* | *1.5* | *-0.2* | *0.6* |
| Range | 2.4 | 1.6 | 2.5 | 1.5 | 0.6 | 3.3 | 1.8 | 0.6 | 1.3 | 4.8 | 2.0 |

**References**

Biggs R, Reyers B, Scholes R (2006) A biodiversity Intactness score for South Africa. S Afr J Sci 102:277–283.

Citizen Surveys (2013) Afrobarometer Round 5 Survey in South Africa Summary of Results. Institute for Democracy in South Africa (IDASA), Cape Town

Cole MJ, Bailey RM, New MG (2016) Spatial disaggregation of national sustainable development indicators: A case study of water use and access in South Africa.

DAFF (2015) Draft Policy Document on the Preservation and Development of Agricultural Land. Department of Agriculture, Foresty and Fisheries, Government of South Africa, Pretoria

DAFF (2010) Status of the South African Marine Fishery Resources 2010. Department of Agriculture, Forestry and Fisheries, Government of South Africa.

DAFF (2012) Status of the South African Marine Fishery Resources 2012. Department of Agriculture, Forestry and Fisheries, Government of South Africa. Pretoria

DAFF (2014) Status of the South African Marine Fishery Resources 2014. Department of Agriculture, Foresty and Fisheries, Government of South Africa, Cape Town

DEA (2011) National list of ecosystems that are threatened and in need of protection. Government Gazette No. 34809, Republic of South Africa

Driver A, Maze K, Rouget M, et al (2005) National Spatial Biodiversity Assessment 2004: priorities for biodiversity conservation in South Africa. Strelitzia 17. South African National Biodiversity Institute, Pretoria

DWA (2013) National Water Resource Strategy June 2013 Second Edition. Department of Water Affairs, Government of South Africa, Pretoria

DWA (2011) Water Reconciliation Strategy Study for the Algoa Water Supply Area. Algoa Reconciliation Strategy (April 2011).

DWA (2012) Water Reconciliation Strategy Study for the Large Bulk Water Supply Systems: Greater Bloemfontein Area. Final Reconciliation Strategy Report (June 2012).

DWA (2009) Water Reconciliation Strategy Study for the KwaZulu Natal Coastal Metropolitan Areas (November 2009). Department of Water Affairs, Government of South Africa

DWAF (2004) National Water Resource Strategy, First Edition. Department of Environmental Affairs and Tourism, Government of South Africa, Pretoria

DWAF (2007) Western Cape Water Supply System Reconciliation Strategy Study. Reconciliation Strategy Final (June 2007). Cape Town

DWAF (2011) Development of a reconciliation strategy for the Amatole Bulk Water Supply System Final Report (March 2008).

DWAF (2009) Vaal River System: Large bulk water supply reconciliation strategy. Second stage reconciliation strategy (March 2009).

Government of the Eastern Cape (2011) Eastern Cape Climate Change Response. Department of Economic Development and Environmental Affairs, East London

NEDLAC (2012) Hydrochlorofluorocarbons (HCFC) Phase Out Plan for South Africa. NEDLAC Trade and Industry Chamber, Ferndale, South Africa

Nel JL, Driver A (2012) South African National Biodiversity Assessment 2011: Technical Report. Volume 2: Freshwater Component. CSIR Report Number CSIR/NRE/ECO/2012/0022/A. Stellenbosch

SDSN (2015) Indicators and a monitoring framework for Sustainable Development Goals - Launching a data revolution for the SDGs. Sustainable Development Solutions Network

Sink K, Holness S, Harris L, et al (2012) South African National Biodiversity Assessment 2011: Technical Report. Volume 4: Marine and Coastal Component. Pretoria

StatsSA (2012a) Statistical release P0301.4 Census 2011. Statistics South Africa, Pretoria

StatsSA (2014) Statistical release P0441 Gross domestic product Annual estimates 2004-2013 Regional estimates 2004-2013 Third quarter 2014. Statistics South Africa, Pretoria

StatsSA (2016a) Excel - Electricity generated and available for distribution 201606. http://www.statssa.gov.za/?page_id=1854&PPN=P4141&SCH=6516. Accessed 28 Aug 2016

StatsSA (2012b) Statistical release P0441 Gross domestic product Annual estimates 2002-2011, Regional estimates 2002-2011, Third quarter 2012. Statistics South Africa, Pretoria

StatsSA (2015) Statistical release P0302 Mid-year population estimates 2015. Statistics South Africa, Pretoria

StatsSA (2016b) Statistical release P0441 Gross domestic product Fourth quarter 2015. Statistics South Africa, Pretoria

Struwig J, Davids YD, Roberts B, et al (2011) Towards a social cohesion barometer for South Africa. Somerset West, South Africa

Tomaschek J, Haasz T, Dobbins A, Fahl U (2012) Energy Related Greenhouse Gas Inventory and Energy Balance Gauteng: 2007-2009. Stuttgart

UN General Assembly (2015) Transforming our world: The 2030 agenda for sustainable development. United Nations General Assembly, New York

UNEP (2016) UNEP Ozone Secretariat Data Access Centre. http://ozone.unep.org/en/data-reporting/data-centre. Accessed 12 Aug 2016

van Niekerk L, Turple JK (2012) South African National Biodiversity Assessment 2011: Technical Report. Volume 3: Estuary Component. CSIR Report Number CSIR/NRE/ECOS/ER/2011/0045/B. Stellenbosch

Western Cape Government (2013) Energy Consumption and CO2e Emissions Database for the Western Cape. Cape Town

YM Mashalaba & Associates (2012) Free State Province Greenhouse Gas Emissions Inventory 2000 - 2012. Bloemfontein, South Africa
